# Deletions of singular U1 snRNA gene significantly interfere with transcription and 3’-end mRNA formation

**DOI:** 10.1371/journal.pgen.1011021

**Published:** 2023-11-02

**Authors:** Mei Wang, An-Min Liang, Zhen-Zhen Zhou, Ting-Lin Pang, Yu-Jie Fan, Yong-Zhen Xu

**Affiliations:** 1 Key Laboratory of Insect Developmental and Evolutionary Biology, Center for Excellence in Molecular Plant Sciences, Chinese Academy of Sciences; Shanghai, China, University of Chinese Academy of Sciences, China; 2 RNA Institute, State Key Laboratory of Virology, Hubei Key Laboratory of Cell Homeostasis, College of Life Sciences, TaiKang Center for Life and Medical Sciences, Wuhan University, Hubei, China; 3 Shanghai Institute of Biological Products, Shanghai, China; U. Wisconsin-Madison, UNITED STATES

## Abstract

Small nuclear RNAs (snRNAs) are structural and functional cores of the spliceosome. In metazoan genomes, each snRNA has multiple copies/variants, up to hundreds in mammals. However, the expressions and functions of each copy/variant in one organism have not been systematically studied. Focus on U1 snRNA genes, we investigated all five copies in *Drosophila melanogaster* using two series of constructed strains. Analyses of transgenic flies that each have a U1 promoter-driven *gfp* revealed that *U1*:*21D* is the major and ubiquitously expressed copy, and the other four copies have specificities in developmental stages and tissues. Mutant strains that each have a precisely deleted copy of U1-gene exhibited various extents of defects in fly morphology or mobility, especially deletion of *U1*:*82Eb*. Interestingly, splicing was changed at limited levels in the deletion strains, while large amounts of differentially-expressed genes and alternative polyadenylation events were identified, showing preferences in the down-regulation of genes with 1–2 introns and selection of proximal sites for 3’-end polyadenylation. *In vitro* assays suggested that *Drosophila* U1 variants pulled down fewer SmD2 proteins compared to the canonical U1. This study demonstrates that all five U1-genes in *Drosophila* have physiological functions in development and play regulatory roles in transcription and 3’-end formation.

## Author summary

Small nuclear RNAs (snRNAs) are the core of the spliceosome, which catalyzes the removal of introns. In metazoan genomes, each snRNA has multiple copies with variant sequences, up to hundreds in mammals, in which many singular copies/variants have shown function and are related to human diseases. However, expressions and functions of all snRNA copies in one organism have remained unknown. U1 snRNA is critical for early intron recognition and spliceosome assembly. The *Drosophila* genome has five U1 genes that generate three U1 variants. Here, we report that the *U1*:*21D* is ubiquitously high in all tissues and developmental stages, *95Ca* is high in the salivary gland and male accessory gland, and *82Eb*, *95Cb*, and *95Cc* are expressed in the ovary and embryo. Deletions of each singular U1-gene cause various developmental defects, demonstrating that none of them are pseudogenes. The absence of a U1-gene results in more serious defects in transcription and 3’-end formation than in pre-mRNA splicing, showing large amounts of expression-changed genes and alternative polyadenylation changes but much fewer changes in alternative splicing. Thus, our findings advance the understanding of *in vivo* functions of all the U1 snRNA genes in one organism.

## Introduction

Coding sequences of eukaryotic genes are interrupted by introns, which must be removed through pre-mRNA splicing that is catalyzed by a large and dynamic RNA-protein complex, the spliceosome [1,2]. Two spliceosomes coexist in most of the higher eukaryotes, the U2-type and the U12-type [3,4]. The U2-type spliceosome, aka the major spliceosome, is composed of U1, U2, U4, U5, and U6 small nuclear RNAs (snRNAs) and over 100 proteins, and catalyzes the removal of >99.5% introns. The U12-type spliceosome, aka the minor spliceosome, is composed of U11, U12, U4atac, U5, U6atac snRNAs, and over 100 proteins, removing much fewer introns [5,6].

snRNA components are the core of the spliceosome, they are bound with proteins to assemble small nuclear ribonucleoproteins (snRNPs) and are responsible for intron recognition, spliceosome assembly, and catalysis [7]. Typically, there are four conserved intronic signals in a pre-mRNA molecule, including the 5’ splice site (5’SS), 3’ splice site (3’SS), a polypyrimidine tract (PPyT), and branch site region (BS). Interactions between the snRNAs and intron signals are critical during the processing of pre-mRNA splicing. For example, 6–8 nucleotides at the 5’-end of U1 snRNA in U1 snRNP base-pairs with the 5’SS of pre-mRNA and initiates the spliceosome assembly on the nascent messenger RNA [8–10], the branch-site recognition motif (4–10 nts) of U2 snRNA base-pairs with the BS region of the U2-type intron that allows for stable assembly of the pre-spliceosome [11–13], and the U2/U6 snRNAs mediate catalysis within the spliceosome [14,15].

U1, U2, U4, U5, U11, U12, and U4atac snRNAs are transcribed by the RNA polymerase II (Pol II), and the U6 and U6atac snRNAs are transcribed by the RNA polymerase III [16]. All the snRNAs are highly structured with multiple intramolecular RNA helices [Reviewed in 6,17]. The Pol II-transcribed mature snRNAs have a 5′-trimethylguanosine cap and a 3′ stem-loop structure that is recognized by the survival of motor neuron (SMN) protein [18,19], while the Pol III-transcribed mature snRNAs have a 5′-γ-monomethylphosphate cap and a 3′ stem-loop for binding of Lsm proteins [20]. Structures and key primary sequences of the spliceosomal snRNAs are highly conserved across species [21–23].

Although spliceosomal snRNAs are present in the unicellular organism *S*. *cerevisiae* as a single copy, they are always present as multiple copies with sequence variations in metazoans, ranging from 5 copies with variations of U1 snRNA in the *D*. *melanogaster* genome to around 200 copies/variants of U1, U2 snRNAs in the mammal genomes, and even to ~1,000 copies/variants of U6 snRNA in the human genome [24–28]. Origins of the multiple copies of spliceosomal snRNAs were proposed from retrotransposons [26,29]. The existence of multiple snRNA copies suggests that they may play indispensable roles in pre-mRNA splicing and other procedures during gene expression in higher eukaryotes.

Initially, the human U1 snRNA gene family was characterized as at least three classes in structure and proposed cycles of gene amplification and transposition in the expansion of multiple copies of these genes [30,31]. Relative expression levels among the spliceosomal snRNA copies/variants are different in tissues and during differentiation and development [23,24,28,32,33]. It has been recently found that snRNAs display distinct expression patterns across five human cell lines and adult and fetal tissues, all U1 snRNA variants are more than 1000-fold less abundant in spliceosomes than the canonical U1, whereas at least 1% of spliceosomes contain a variant of U2, U4 or U5 snRNA, suggesting that each spliceosome is composed by different variants from the five snRNAs [28]. In addition, the maturation and stability of snRNA variants can be selectively regulated by TOE1, an enzyme involved in the 3’-end processing of snRNAs [34].

Pre-mRNA splicing can generate multiple mRNA isoforms from one gene through alternative splicing (AS), which significantly increases the genome and proteasome complexities and regulates gene expression during cell differentiation and development [35–37]. Mutations or deletions in the spliceosomal components and intronic splicing signals are key factors causing developmental defects and human diseases [38–42]. Much evidence has shown that mutations of the spliceosomal snRNAs also greatly affect the correct occurrence of pre-mRNA splicing and result in defective development and diseases [43–46]. For example, the first in vivo study about snRNA variants shows that a mouse model carried mutations in *Rnu2-8*, one of the U2 genes, exhibits ataxia and neuron degeneration, suggesting that the expression of U2 genes is spatially and temporally regulated [45].

U1 snRNA (U1), one of the most abundant non-coding RNAs in cells, is highly conserved in length (164 nt) and secondary structure except in *S*. *cerevisiae* which has additional long loops [47]. U1 also has been found in the regulation of transcription by interacting with the SAGA complex [48], of chromatin retention through binding with IncRNAs [49], and of the mRNA 3’-end polyadenylation through telescripting [19,50,51]. Knockout or inhibition by antisense morpholino oligonucleotides (AMO) of U1 genes results in an increased accumulation of unspliced pre-mRNAs and premature cleavage/polyadenylation [51,52], and the low doses of U1 AMO increase migration and invasion of cancer cells [52]. Abnormal U1 snRNAs have been detected in patients’ brains with Alzheimer’s disease [53], and Tau can bind to the protein components of U1 snRNP in *Drosophila* leading to splicing disorder and neuronal degeneration [54], suggesting that U1 snRNP may be a potential pathogenesis and target of Alzheimer’s disease [55]. Recently, a highly recurrent hotspot mutation (r.3A>G) of U1 snRNA was found in the Sonic hedgehog (SHH) medulloblastomas, leading to significantly disrupted RNA splicing and an excess of 5’ cryptic splicing events in tumors [44].

Expression of snRNA variants in one organism has been studied in the fly, mouse, and human [22,24,27,28]; however, expression of each snRNA copy in one organism has not been identified due to the same sequences of many copies, and the *in vivo* functional analysis of all copies/variants in one organism has not been systematically studied. Taking advantage of the powerful genetic systems, here we focused on the U1 snRNA and systematically investigated all five *Drosophila melanogaster* U1 genes. We constructed two series of U1-gene strains, one being transgenic strains of U1 promoter-driven *gfp* that allows for detection of each U1-gene’s expression in various developmental stages and tissues, and the other being CRISPR/Cas9-mediated precise U1-gene deletion strains that allow for the investigation of phenotypes and effects on multiple RNA processing steps, including transcription, splicing, and 3’-end formation. Further *in vitro* pulldown assays reveal that the *Drosophila* U1 variants have different activities in binding with SmD2 proteins.

## Results

### *U1*:*21D* is ubiquitous, while the other four U1 genes are stage or tissue-specific

**Fig 1:**
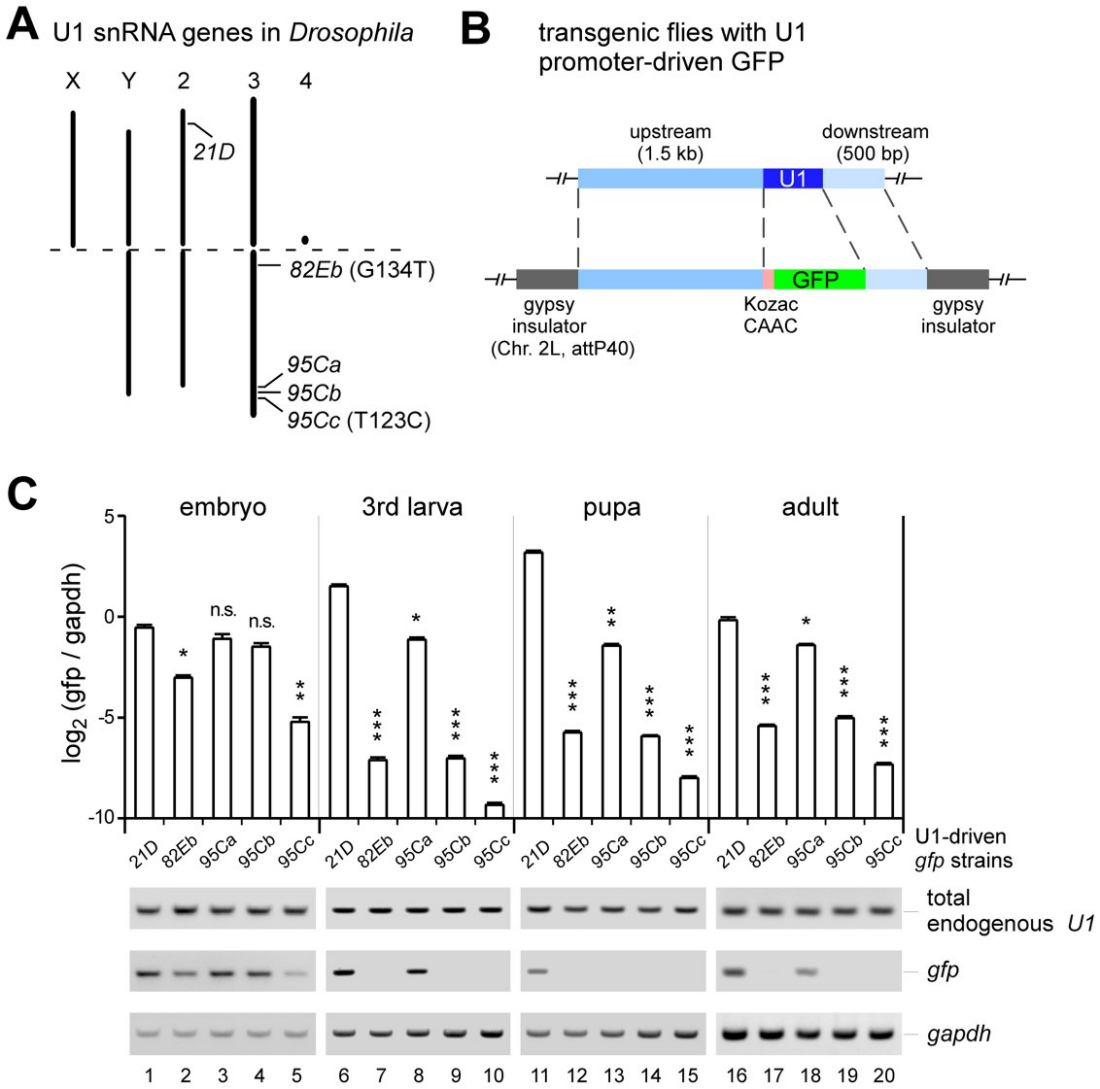
Expression of singular U1 snRNA genes during the *Drosophila* developmental stages. (**A**) Schematics of genomic loci of the five *Drosophila* U1 snRNA genes. (**B**) Strategy for construction of transgenic flies of the five U1-driven *gfp*. (**C**) Quantitative RT-PCR analyses of the *gfp* mRNAs that are driven by each singular U1-genes at the four *Drosophila* developmental stages. Regular RT-PCRs were also performed for the detection of the total endogenous U1 snRNAs. ΔCt = Ct_U1-driven *gfp*_—Ct_*gapdh*_, all Ct values were less than 40 cycles. All data are shown as means ± SEM, and a *t*-test was used for statistical analysis. *, p < 0.05; **, p < 0.01; ***, p < 0.001.

Compared to hundreds of copies in mammals, U snRNA genes are in the single digits in *D*. *melanogaster*, allowing us to investigate the functions of each copy (Fig 1A and S1A Fig). There are five copies of U1-genes in the fruit fly, in which the *U1*:*21D* gene is located on chromosome 2, while the other four (*82Eb*, *95Ca*, *95Cb*, and *95Cc*) are on chromosome 3R (Fig 1A). Sequence variations of the five U1 snRNAs are small. U1 snRNA transcribed from *21D*, *95Ca*, and *95Cb* are the same and annotated as the canonical U1; the *95Cc* has a position 123 U-to-C variation, and the *82Eb* has a position 134 G-to-U variation (Fig 1A). Both variation sites are in or very close to the Sm protein binding site, which is critical for the assembly of spliceosomal snRNPs (S1B Fig). We aligned the upstream and downstream 1 Kb sequences of the five U1 genes. Although it is hard to determine the copy-specific promoter motif sequences, we found that three U1 genes, *21D*, *82Eb*, and *95Ca*, have closer sequence similarities than the other two U1 genes, *95Cb*, and *95Cc* (S2 Fig). To accurately distinguish the expression of each U1 gene, we constructed five transgenic fly strains; each has a respective U1-promoter-driven *gfp* followed by its U1 termination region, which was inserted into the attP40 site on chromosome 2L (Fig 1B). The total endogenous U1 snRNAs in the five transgenic strains were at similar levels (Fig 1C bottom), suggesting that insertion of the transgenic U1-driven *gfp* did not affect the expression of the endogenous U1 snRNAs. Therefore, the expression of each U1 gene can be detected by the GFP signals from its transgenic fly. Using RT-qPCR, we first measured *gfp* mRNA levels in samples from the four developmental stages, including the embryo, the 3^rd^ instar larva, the pupa, and the adult (Fig 1C). In comparison to *gapdh*, all five U1-*gfp* were relatively abundant at the embryonic stage, in which *21D-*, *95Ca-*, and *95Cb*-*gfp* were at similarly high levels, and *82Eb-* and *95Cc*-*gfp* were relatively lower (Fig 1C lanes 1–5). In contrast, only *21D*- and *95Ca*-*gfp* were abundant at the 3^rd^ instar larva and adult stages, and only *21D-gfp* was abundant at the pupa stage; the other three U1*-gfp*s were at very lower levels in those three stages (Fig 1C lanes 6–20).

**Fig 2:**
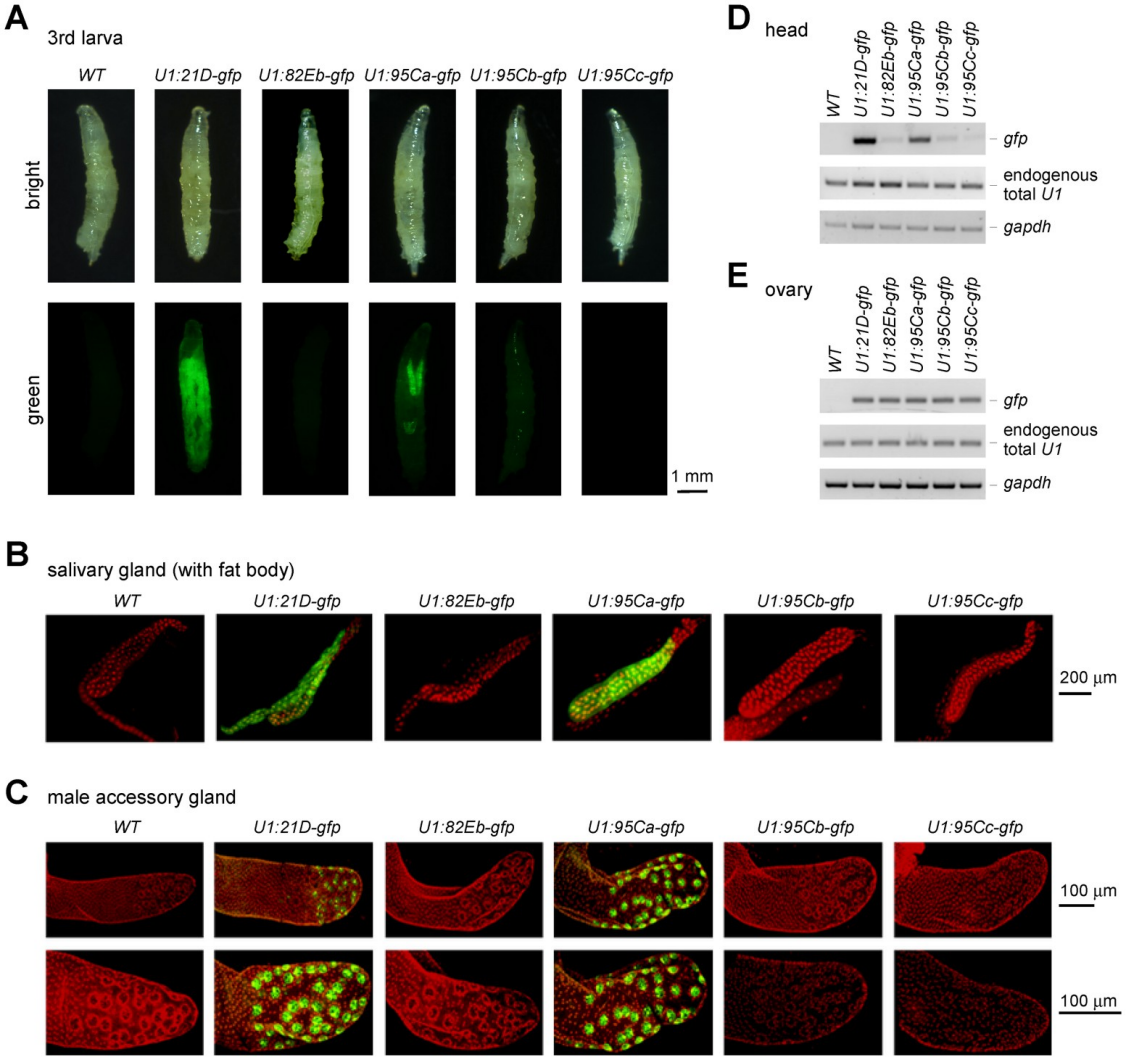
Expression of singular U1 snRNA genes in *Drosophila* tissues. Fluorescent signals of U1-gene-driven GFPs were detected for larvae by stereoscope imaging (**A**), for the salivary gland (**B**), and male accessory gland (**C**) by confocal imaging. Scales are indicated; green, GFP; red, DAPI. (**D**) Detection of the U1-driven *gfp* mRNAs in the head by RT-PCR. (**E**) Detection of the U1-driven *gfp* mRNAs in the ovaries by RT-PCR.

Taking advantage of the GFP signal, we then detected tissue specificities of U1 genes by fluorescent microscope. At the 3^rd^ larva stage, the GFP signal from the *21D*-*gfp* was detected ubiquitously all through the body and the expression of *95Ca*-*gfp* was around the salivary gland region, while the other three U1s did not show detectable signals (Fig 2A). Furthermore, we dissected the salivary gland and found that the *21D*-*gfp* strain showed strong green fluorescent signals in the salivary gland and its associated fat tissues, whereas the *95Ca-gfp* strain showed strong signal only in the salivary gland, and the other three U1s showed no detectable signals (Fig 2B). Similarly, samples from the male accessory gland and head of the *21D-* and *95Ca-gfp* transgenic flies exhibited strong GFP (or *gfp* mRNA) signals, but those tissues from the other three *U1-gfp* strains did not (Fig 2C and 2D). GFP signals in the accessory gland are exclusively condensed in the secondary cells interspersed at the distal tip of each accessory gland lobe [56]. No GFP signals were detected in the main cells or other cells (Fig 2C). Fluorescent signals from adults were also consistent with the above RT-qPCR results, both females and males of the *21D-gfp* strain exhibited the strongest GFP signals (S3A Fig). Interestingly, the *U1-gfp* mRNAs were at similar levels in ovaries from all five transgenic strains (Fig 2E), as well as their fluorescent signals (S3B Fig). This is consistent with the *gfp* mRNA levels in embryos (Fig 1C), implying that transcription of all five U1s are at roughly similar levels from the egg in the ovary to the embryonic stage.

We also retrieved ChIP-seq data of *Drosophila* transcription factors (TFs) from the ChIP-Atlas [57]. Defined by the AnimalTFDB [58], we obtained 45 TFs have binding peaks on the five U1-genes (S4A Fig). Interestingly, we noticed that the *95Ca* gene is strongly co-immunoprecipitated by Trl in the salivary gland (S4B Fig). Trl is a GAGA transcription factor that is involved in chromatin modification [59]; its strong binding might be the reason for the high expression of *95Ca* promoter-driven *gfp* in the salivary gland (Fig 2B).

Taken together, we conclude that *21D* is the major U1 gene in expression, which is ubiquitously and highly transcribed in all *Drosophila* tissues and developmental stages; *95Ca* is the secondly abundant U1 gene in expression, which has specificities in the salivary gland and male accessory gland; whereas other three U1s, *82Eb*, *95Cb* and *95Cc*, are only expressed relatively high in ovary and embryo.

### Singular U1-gene deletions result in inconsistent changes in the remaining total U1 snRNAs

**Fig 3:**
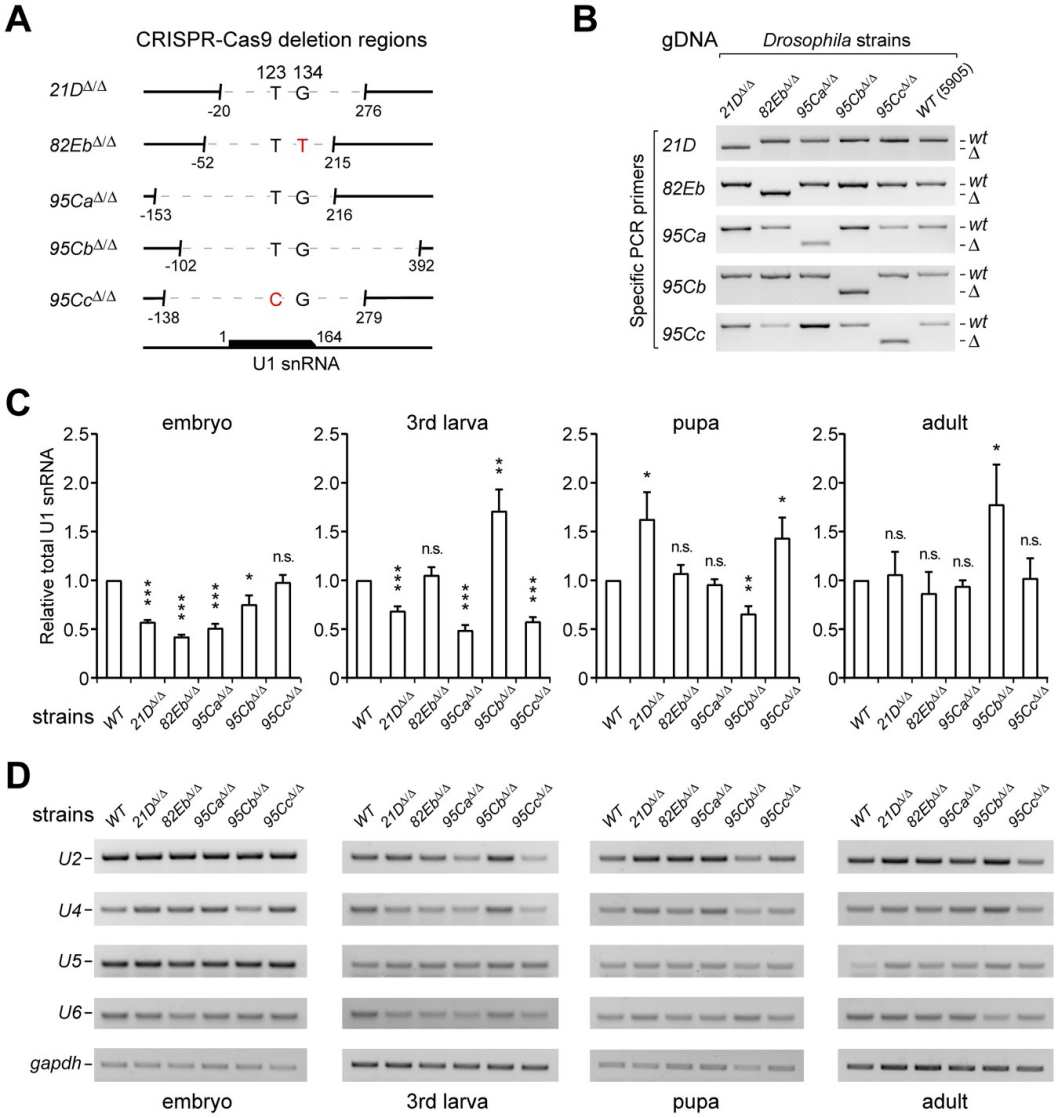
Deletions of each U1-gene result in fluctuated levels of snRNAs during the *Drosophila* developmental stages. (**A**) Deletion regions of the five U1-genes by a CRISPR/Cas9 mediated knock-in system. Dashed lines, deletion regions. Positions and variation sites are indicated. (**B**) Confirmation of the five U1-gene deletion strains by genomic PCRs using specific primers. (**C**) The remaining total endogenous U1 snRNAs in the five U1-gene deletion strains fluctuated in the four *Drosophila* developmental stages. Total U1 snRNAs were detected by real-time RT-PCR using common primers. (**D**) Detection of other spliceosomal snRNAs in the U1-gene strains using regular RT-PCR. ΔCt = Ct_U1 snRNA_—Ct_*gapdh*_, all Ct values were less than 40 cycles, normalized by values in the *WT* strain and compared by *t*-test. All data are shown as means ± SEM. *P < 0.05, **P < 0.01, ***P < 0.001.

To investigate the function of each U1 gene, we further constructed deletion strains of each U1 through CRISPR/Cas9-mediated genome editing, in which the region containing promoter, transcript, and termination were precisely deleted, depending on the selected suitable PAM sequences for guide RNAs (Fig 3A). Screened by genomic PCRs and Sanger sequencing, the five U1-gene deletion strains were successfully obtained (Fig 3B). We then examined how much total U1 snRNAs are left when one U1-gene is deleted during the four developmental stages. Deletion of the ubiquitously-expressed *21D* (*21D*^*Δ/Δ*^) resulted in a significant decrease of the remaining total U1 snRNAs in embryo and larva, but no obvious changes in adult, and even resulted in a significant increase in pupa (Fig 3C). Similarly, deletion of *95Ca*, the second major U1 gene, resulted in a decreased total U1 snRNAs in embryo and larva, but no obvious changes in pupa or adult. Oddly, among the other three less-expressed U1 genes, the *95Cb*^*Δ/Δ*^ strain exhibited a significant increase of remaining total U1 snRNAs in larva and adult and no obvious changes in embryo or pupa, whereas the *82Eb*^*Δ/Δ*^ strain showed a dramatic decrease of the total U1s in the embryo and no obvious changes in other three stages (Fig 3C). We also observed fluctuant changes of other spliceosomal snRNAs in these five U1-gene deletion strains (Fig 3D), consistent with a recent study from mammals [60]. The most obvious changes include: i) the U2 and U4 snRNAs were significantly increased at the pupa stage in the deletion of *21D*, *82Eb*, and *95Ca* strains, but were decreased at the larva stage in the deletion of *95Cc* strain; ii) the fluctuant changes were smaller at embryo stage than at the other three stages. Taken together, these results imply that the expression of the five U1 genes would be controlled by an unknown regulatory network, in which regulation of other spliceosomal snRNAs might be also involved.

### Singular U1-gene deletion strains have defects in development and mobility

**Fig 4:**
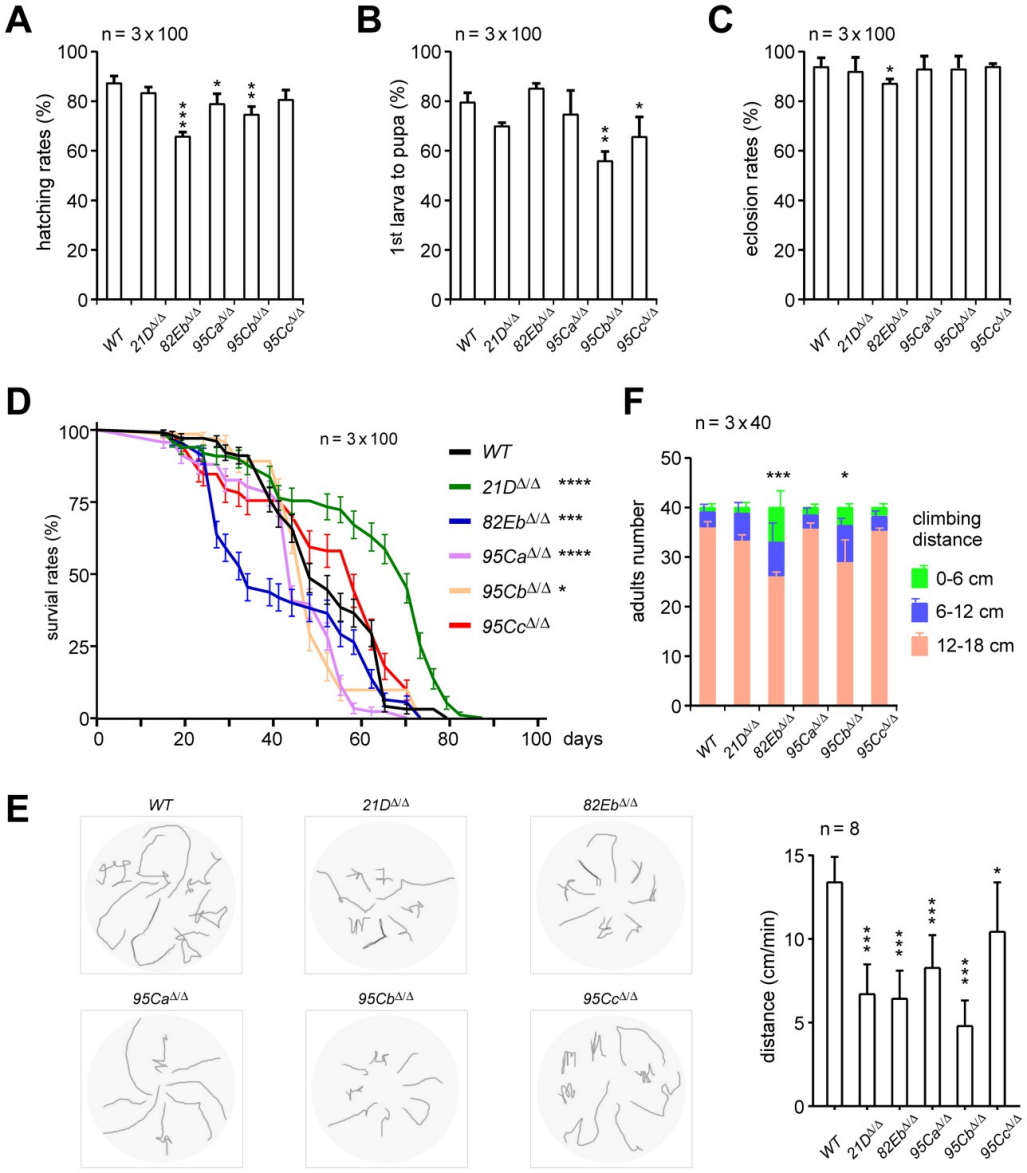
U1-gene deletion strains exhibit various defective phenotypes in *Drosophila* development and mobility. Investigation of the developmental phenotypes for the five U1-gene deletion strains, including hatching rates (**A**), pupation rates (**B**), eclosion rates (**C**), and lifespans (**D**). Detection of mobility defects in larvae locomotion **(E**) and adults climbing (**F**) for the singular U1-gene deletion strains. All data are shown as means ± SEM. *P < 0.05, **P < 0.01, ***P < 0.001.

Firstly, we examined and found that none of the five deletion strains were lethal or sterile, suggesting that no singular U1-gene is essential for the survival of *Drosophila*. We then investigated their phenotypes in viability, morphology, and mobility, including rates of hatching, pupation and eclosion, lifespan, larva locomotion, and adult climbing. To our surprise, deletion of the ubiquitously-expressed U1-gene, *21D*^*Δ/Δ*^, was not the most affected strain with defective phenotypes and even had an elongated lifespan of adults (Fig 4). In contrast, deletion of the two less-expressed U1s, *82Eb*^*Δ/Δ*^, and *95Cb*^*Δ/Δ*^, exhibited more seriously defective phenotypes, especially the *82Eb*^*Δ/Δ*^ (Fig 4). For example, both the *82Eb*^*Δ/Δ*^ and *95Cb*^*Δ/Δ*^ strains had significantly decreased hatching rates, shorten lifespan, and impaired larva locomotion and adult climbing (Fig 4A, 4D, 4E and 4F), and *82Eb*^*Δ/Δ*^ also had a slightly decreased rate of eclosion (Fig 4C). Notably, larvae from all the U1-deletion strains had significantly impaired locomotion abilities (Fig 4E), while only adults from the *82Eb*^*Δ/Δ*^ and *95Cb*^*Δ/Δ*^ strains exhibited defects in climbing (Fig 4F). Although many of the phenotypic effects of deleting a single U1 gene are minimal, these results demonstrate that each of the five *Drosophila* U1-genes is functionally involved in various aspects of development and that the extents of their contributions are not fully correlated with their relative expression levels.

In addition, homozygotic double-deletion of *21D* and *95Ca* genes, the most two abundant U1-genes detected by the above U1-driven *gfp* signals, resulted in the lethality of *Drosophila* at its embryonic stage; whereas their heterozygotic double-deletion and homozygotic double-deletion of other U1-gene combinations were still viable (S4C and S4D Fig).

### Transcription is seriously affected, especially in the *82Eb*^*Δ/Δ*^ strain

**Fig 5:**
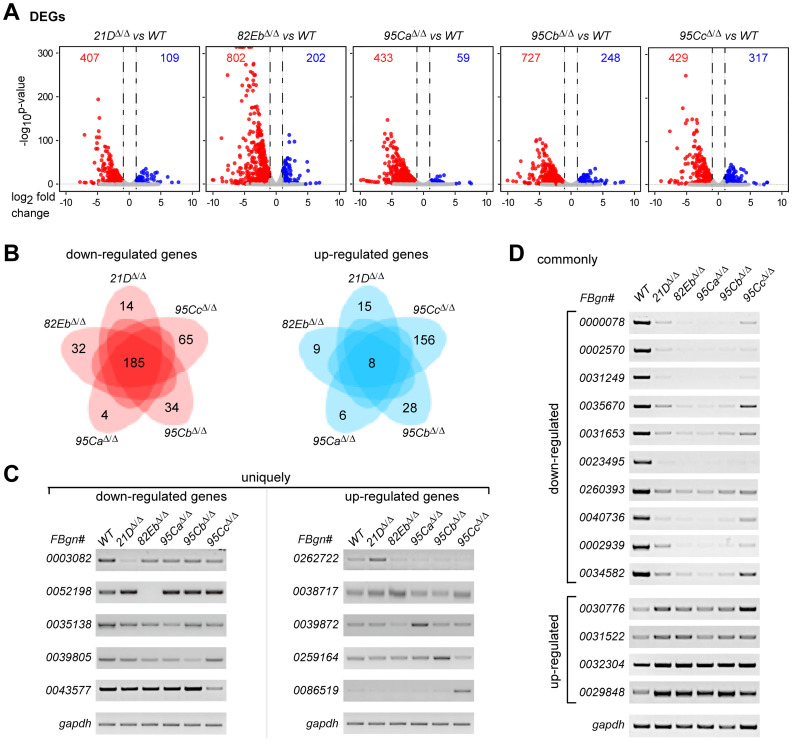
Transcriptions are dramatically affected in the singular U1-gene deletion strains. (**A**) Differently expressed genes (DEGs) in the five U1-gene deletion strains compared to the *WT*. DEGs were analyzed from the RNA-seq data of the six fly strains. Numbers of the down- and up-regulated genes are indicated in red and blue, respectively. (**B**) Commonly and uniquely regulated genes in the five U1-gene deletion strains. DEGs are grouped into down-regulated and up-regulated. (**C**) Validation of the uniquely down- and up-regulated genes by RT-PCR. (**D**) Validation of the commonly down- and up-regulated genes by RT-PCR.

We further performed Illumina RNA-seq of the five deletion strains using their 3^rd^ instar larvae (S1 Table). Bioinformatic analyses showed that there were at least 450 differentially expressed genes (DEGs) in each strain. The *82Eb*^*Δ/Δ*^ and *95Cb*^*Δ/Δ*^ strains exhibited the most significant changes in expression, each having ~1,000 DEGs; however, the *21D*^*Δ/Δ*^ and *95Ca*^*Δ/Δ*^ strains exhibited relatively less, each having ~500 DEGs (Fig 5A and S2 Table). In comparison to the *WT*, most of the affected genes were down-regulated in the deletion strains. For example, the *82Eb*^*Δ/Δ*^ exhibited 802 down-regulated genes versus 202 up-regulated genes and the *95Ca*^*Δ/Δ*^ exhibited 433 versus 59, suggesting that *in vivo* deletion of a U1-gene resulted in a majorly decreased activity of transcription. Further analyses found that there are 185 and 8 DEGs were commonly down-regulated and up-regulated, respectively; many of the DEGs in each deletion strain were unique, especially the *95Cc*^*Δ/Δ*^ has 221 uniquely expression-affected genes (Fig 5B and S5A Fig). By a principal component analysis (PCA), we found that the *82Eb*^*Δ/Δ*^ and *95Ca*^*Δ/Δ*^ strains are closer in gene expressions than the other three KO strains (S5B Fig). To experimentally validate these findings, we randomly picked one of the uniquely down- and up-regulated from each strain (Fig 5C), ten of the commonly down-regulated, and four of the commonly up-regulated genes (Fig 5D). RT-PCR assays confirmed that expression changes of those genes in each U1-deletion strain are consistent with the above bioinformatic analyses.

Through GO analyses, we first found that the expression-affected genes in the *21D*^*Δ/Δ*^ strain did not exhibit enriched functional pathways, whereas the other four U1-deletion strains did, being consistent with the ubiquitous expression of *21D* and milder phenotypes of its deletion (S5C Fig). Secondly, the expression-affected genes in the other four U1-deletion strains are significantly enriched in cuticle development, defense response, and muscle cell development (S5C Fig). This is consistent with their defective phenotypes in metamorphosis and mobility, suggesting that those DEGs may contribute to the defective phenotypes in each U1-deletion strain (Fig 4). Thirdly, among the commonly down-regulated genes in the five deletion strains, they were enriched in serine-type peptidases, hydrolases, lipases, and transferases (S5D Fig). By analyses of the transcriptome data from multiple tissues on the Flybase, we found that most of the commonly down-regulated genes are highly expressed in the midgut (S5E Fig), implying an interesting regulation of these genes by all five U1 genes in *Drosophila*.

### Singular U1-gene deletion changes *in vivo* splicing at a limited level

**Fig 6:**
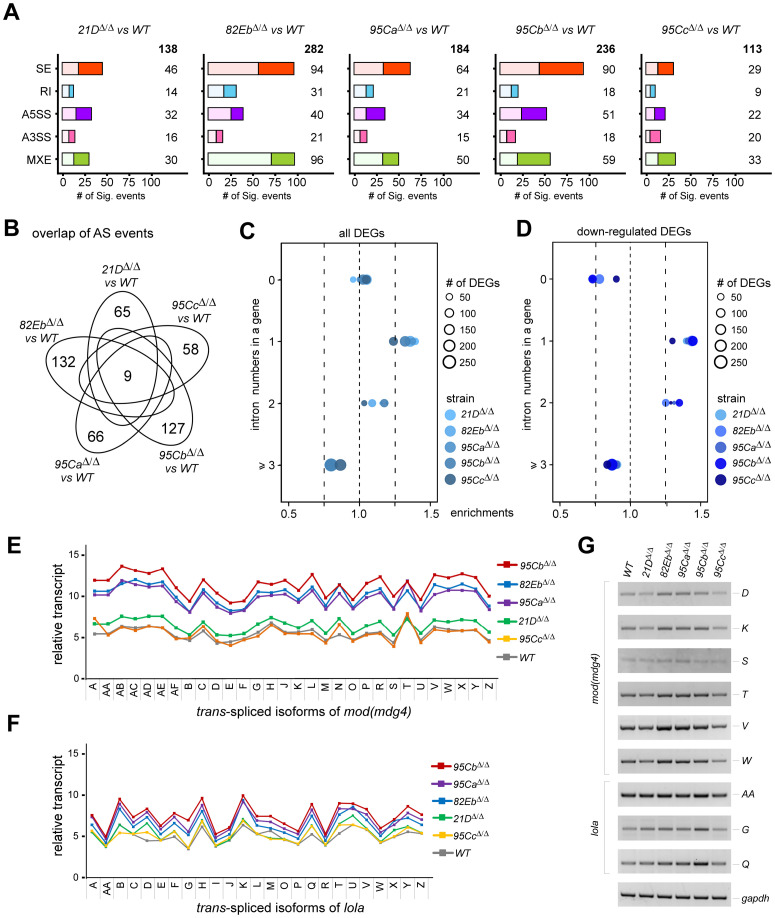
Singular U1-gene deletion results in mild effects on alternative splicing and *trans*-splicing in *Drosophila*. (**A**) Differently changed AS events in the five U1-gene deletion strains compared to the *WT*. AS events are grouped into five types, including skipped exon (SE), retained intron (RI), mutually exclusive exons (MXE), alternative 5’SS (A5SS), and alternative 3’SS (A3SS). The numbers of changed AS events are indicated. Dark boxes, *ΔPSI* > 0.05 events, light boxes, *ΔPSI* < -0.05 events. (**B**) Commonly and uniquely changed AS events in the five U1-gene deletion strains. Analyses of all the DEGs (**C**) and the down-regulated DEGs (**D**) in the U1-gene deletion strains exhibit a significant enrichment of genes with 1~2 introns. Analyses of the classical *trans*-spliced genes *mod(mdg4)* (**E**) and *lola* (**F**) indicate that three of the U1-gene deletions strains have mildly increased *trans*-splicing activity. (**G**) Validation of changed *trans*-splicing activities in the five U1-deletion strains by RT-PCR. Isoforms of the two *trans*-spliced genes are named according to annotations on Flybase.

We then analyzed changes in pre-mRNA splicing in the deletion strains. To our surprise, fewer differential alternative splicing events (DASs) were identified in the singular U1-gene deletion strains (Fig 6A and S3 Table), compared to mutations or deletions of other splicing factor in *Drosophila*, such as the *Sf3b1* mutants, *U12* and *U6atac* deletion strains, which caused more than one thousand changed AS events but less than five hundred DEGs [42,61]. Consistent with the extent of defective phenotypes, the *82Eb*^*Δ/Δ*^ and *95Cb*^*Δ/Δ*^ strains also exhibited the most changed DASs (Fig 6A and S6A Fig); however, each only had 282 and 236 events respectively, unlike more than 1,000 DEGs in these two strains (Fig 5A). Similarly, only 9 DASs were commonly changed in the five U1-deletion strains (Fig 6B and S6B Fig). These results revealed that deletion of one U1-gene has limited effects on pre-mRNA splicing, at least in quantities.

Since U1 snRNA is one of the essential components of spliceosome for intron recognition, we then asked whether those DEGs had features connected with splicing or other characters. First, we grouped DEGs by the numbers of containing introns and found that DEGs with 1–2 introns had been significantly enriched in the five deletion strains, while intron-less and multiple-intron-containing DEGs were not (Fig 6C and S6C Fig), and the enrichments were more obvious in the down-regulated DEGs (Fig 6D). Similarly, DEGs in a short length (< 2 Kb) and DEGs with short introns (< 0.5 Kb) were also significantly enriched; while DEGs with different GC contents did not (S7 Fig). These data suggest that the transcription of a gene would be easier paused at its early transcribed region with a U1-binding site when a U1-gene was deleted.

We also analyzed isoforms from two classical *trans*-spliced *Drosophila* genes, *mod(mdg4)* and *lola*, which both have a critical sequence that strongly binds with U1 snRNP in their last common introns for regulation of *trans*-splicing [62]. Validated by RT-PCR analyses, three deletion strains, *82Eb*^*Δ/Δ*^, *95Ca*^*Δ/Δ*^ and *95Cb*^*Δ/Δ*^, showed increased *trans*-splicing activities of these two genes; while slightly changed in the *21D*^*Δ/Δ*^ and *95Cc*^*Δ/Δ*^ (Fig 6E, 6F and 6G). These results imply that expression of the three U1-genes (*82Eb*, *95Ca*, and *95Cb*) would have similar tissue-specificity with expression of the two *trans*-spliced genes.

### Preferential selection of proximal sites for 3’-end formation in U1-gene deletion flies

**Fig 7:**
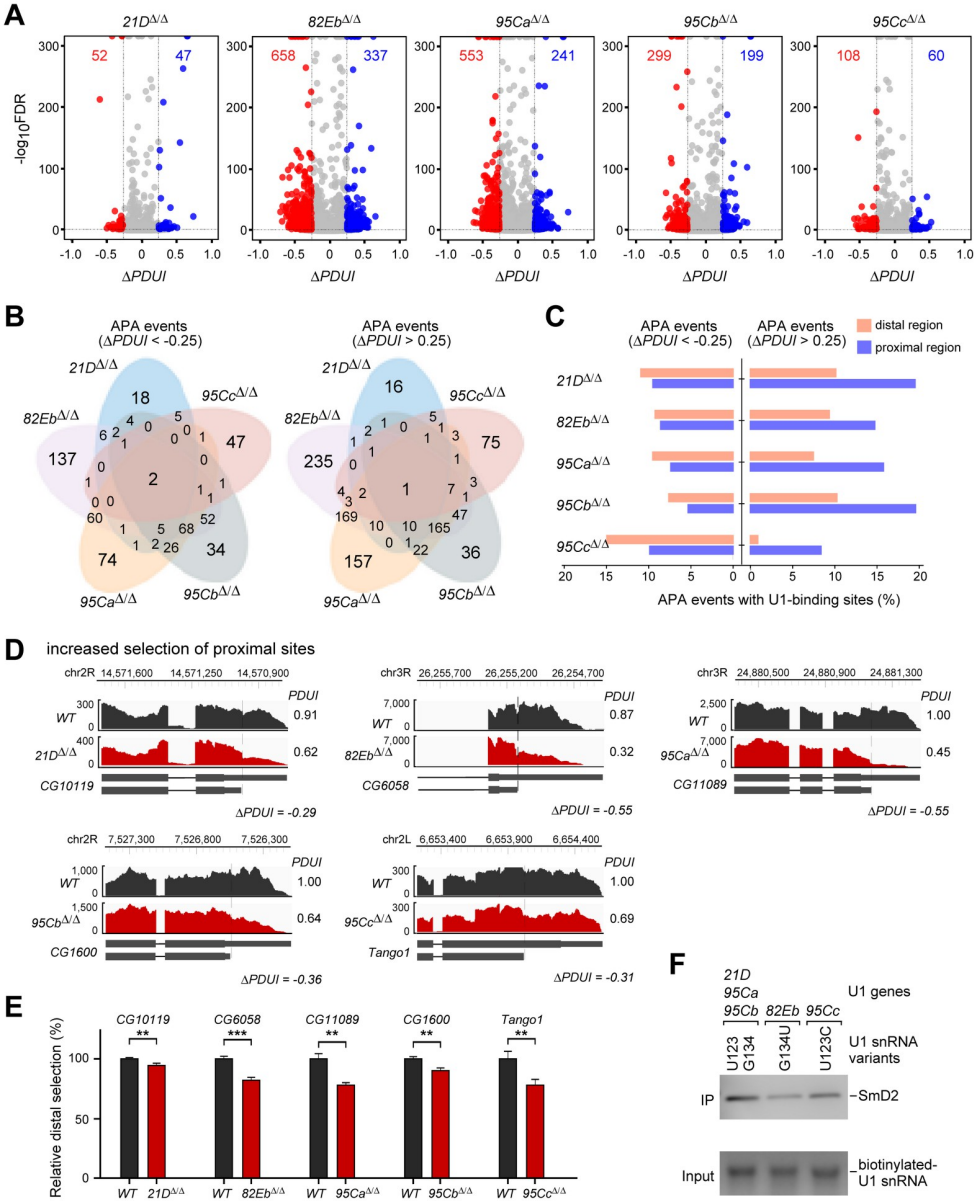
Deletions of singular U1-gene exhibit a preferential selection of proximal sites during the 3’-end formation. (**A**) Differently changed alternative polyadenylation (APA) events in the five U1-gene deletion strains. The number of APA events is indicated. Blue dots, *ΔPDUI* > 0.25 (favor distal sites), red dots, *ΔPDUI* < -0.25 (favor proximal sites). (**B**) Overlapping of the APA events in the five U1-deletion strains. (**C**) Ratio analyses of the U1-binding sites in the proximal and distal regions of the APA events. (**D**) Selected APA events from the five U1-deletion strains, more examples are listed in S8 Fig. (**E**) Validation of the APA events by RT-qPCR. The amplification of the long 3’-UTR region represents the selection of distal site, and the amplification of the common region of all the 3’-UTR isoforms represents both selections (distal + proximal). The relative distal selection is presented by the ratio of distal-to-common in each strain, which is normalized by the value in *WT*. All data are shown as means ± SEM. *, p < 0.05; **, p < 0.01; ***, p < 0.001. (**F**) The two U1 snRNA variants, G134U and G123C, pulled down fewer SmD2 proteins than the canonical U1 snRNA from the lysate of *Drosophila* S2 cells. U1 snRNAs are *in vitro* transcribed and biotin-labeled.

Since U1 snRNP has been reported in the regulation of 3’-end cleavage/polyadenylation for mRNAs [51,52], we then analyzed alternative polyadenylation (APA) events in the U1-deletion strains and found that the *82Eb*^*Δ/Δ*^, *95Ca*^*Δ/Δ*^, and *95Cb*^*Δ/Δ*^ strains had large amounts of APA events (995, 794 and 498, respectively), while the *21D*^*Δ/Δ*^ and *95Cc*^*Δ/Δ*^ had much less (Fig 7A and 7B and S4 Table). Interestingly, there was more selection of proximal sites than distal sites for the 3’-end polyadenylation in the *82Eb*^*Δ/Δ*^, *95Ca*^*Δ/Δ*^, and *95Cb*^*Δ/Δ*^ strains, showing 658 *vs* 337 in *82Eb*^*Δ/Δ*^, 553 *vs* 241 in *95Ca*^*Δ/Δ*^, and 299 *vs* 199 in *95Cb*^*Δ/Δ*^ (Fig 7A, cf. dots red to blue). These results suggest that the presence of these three U1-genes favors facilitating the selection of distal sites for the mRNA 3’-end formation. Since the overall U1 level in the *82Eb*^*Δ/Δ*^ larva was not significantly changed (Fig 3C) but it showed the most changed APA events (Fig 7A), these identified APA events would be due to lacking a specific U1 gene.

The APA events could be divided into at least two groups: changes in the 3’-UTR regions and in the CDS exon/intronic regions (S7D Fig), which are regulated by different mechanisms. In this study, our further focus is on the APA events changed in the 3’-UTR regions. The preferential selection of proximal sites in each of the U1-KO strains is generally consistent with the telescripting model [50], in which the decreased U1 level results in the selection of the proximal polyA sites. To address how the distal polyA sites are activated in this study, we searched U1-binding sites nearby the sequences of all the APA sites (+/- 200 nts) and found that for the proximal-site activated APAs, there are nearly equal amounts of U1-binding sites in the nearby sequences of the proximal and distal regions. However, for the distal-site activated APAs, there are significantly more U1-binding sites in the nearby sequences of their proximal regions than in their distal regions (Fig 7C). This result implies that a U1-binding site at the proximal region but not at the distal region would promote the selection of the proximal site for polyadenylation in the *WT*. However, this promotion would turn to select the distal site when a U1-gene was deleted. To validate the APA events, we performed RT-qPCR to quantitatively detect the ratio of distal site selection and found that 14 out of the selected 20 events display significant changes same as the results of bioinformatic analyses (Fig 7D and 7E and S8 Fig), including 9 events with decreased distal selection and 5 events with increased distal selection in the U1-KO strains.

Furthermore, we performed pull-down assays to address whether the U1 snRNP assembly is different between the U1 snRNA variants using an *in vitro* transcription system and an antibody against SmD2, one of the seven Sm ring proteins that are important for snRNP assembly. In comparison to the canonical U1 snRNA sequence (transcribed from *21D*, *95Ca*, and *95Cb*), both the G134U variant (from *82Eb*) and G123C variant (from *95Cc*) pulled down fewer SmD2 proteins (Fig 7F). This result is consistent with the two sites located in (G134) and near (G123) the binding site for Sm proteins in U1 snRNA (S1B Fig), implying that the U1 variants, especially the *82Eb* (G134) has decreased Sm ring association, could form a less stable U1 snRNP overall and consequentially affect the binding of other U1 proteins and the half-life of U1 snRNA.

## Discussion

Spliceosomal snRNA genes are presented in multiple copies with variations in most metazoans [24–27], and a few snRNA copies/variants and their mutations have been recently shown important roles in development and diseases [44,53–55]. However, due to their large copy numbers in higher eukaryotes and small differences in sequence between variants, systematic investigation on all copies of any certain snRNA gene in an organism has not been performed yet. Therefore, understanding the functions of each copy of the snRNA gene is limited. In this study, we systematically studied the expression and function of all five U1 genes in *D*. *melanogaster*.

### All five U1-genes are functional and contribute to *Drosophila* development

Through the detection of *gfp* mRNAs and GFP fluorescence of U1-driven transgenic strains, we identified that the *U1*:*21D* copy is expressed in all the tested fly developmental stages and tissues, and the other four U1-genes have expression specificities during development and tissues. Furthermore, all five U1-gene deletion strains exhibit defects to various extents in the fly development and/or mobility, suggesting that each of the five U1-genes has physiological functions in *Drosophila*, and none of them is a pseudogene. Notably, among the five deletion strains, deletion of *U1*:*82Eb* exhibits the most serious defects in nearly all the tested phenotypes, indicating that the *U1*:*82Eb* copy is important for the fly development although its expression level is always lower than the *U1*:*21D* copy and undetectable at many developmental stages and tissues. However, among double-deletion stains, only deletion of *U1*:*21D* and *U1*:*95Ca* together, the two most abundant U1-genes, result in the lethality of *Drosophila*. These puzzles would be solved by future-developed single-cell sequencing if it could detect mRNAs and snRNAs at the same time in a cell, and let us understand more details about the expression and function of each U1-gene in different cell types.

### Each U1-gene is critical for transcription and the 3’-end formation

**Fig 8:**
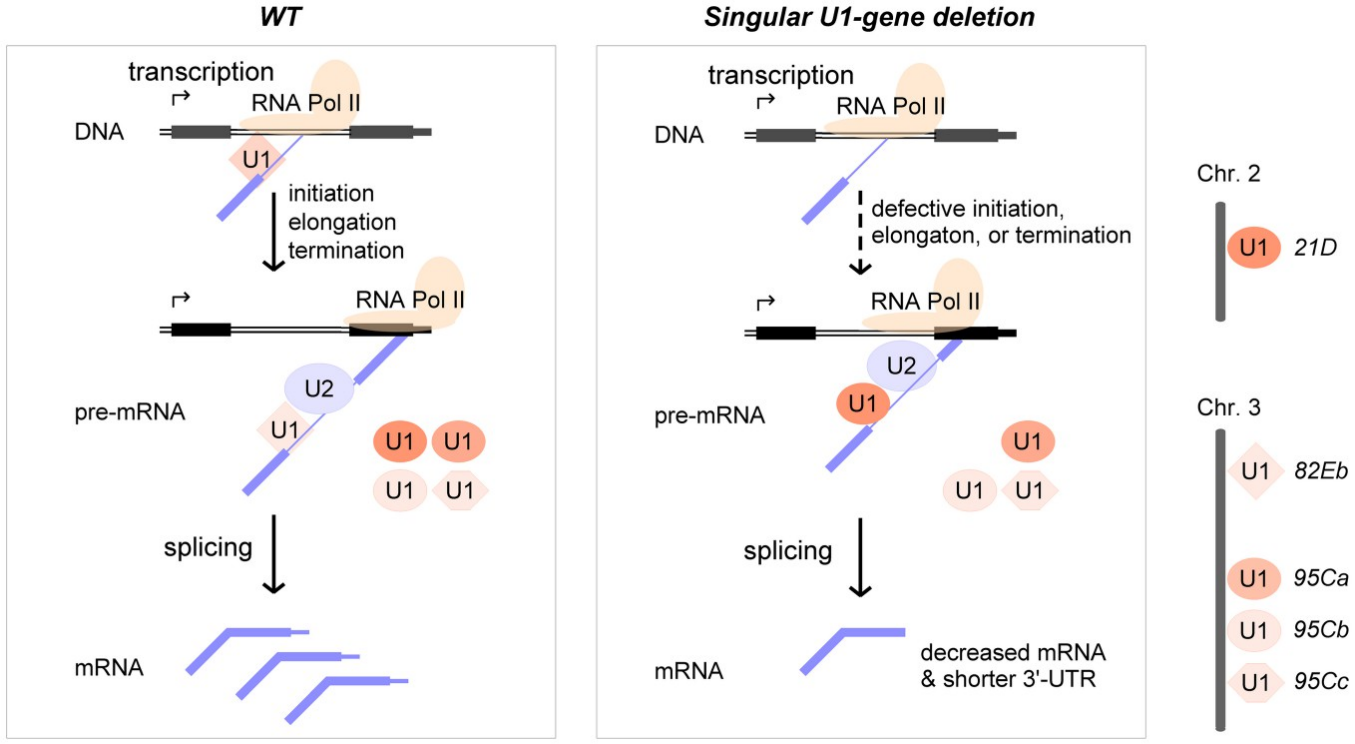
Deletions of singular U1 snRNA gene significantly interfere with transcription and 3’-end mRNA formation rather than pre-mRNA splicing. Left, in the *WT Drosophila*, all five U1-genes have functions in pre-mRNA splicing in a transcription-coupled way. Right, in the singular U1-gene deleted *Drosophila*, transcriptions of many genes are defective due to lacking specific U1-binding at the 5’SS of pre-mRNA, resulting in decreased mRNA levels and shorter 3’-UTRs. However, the pre-mRNA splicing could be compensated by other U1s. For clarity, only one copy of U1-gene is illustrated in each case. U1-genes are indicated in ovals for the canonicals and non-ovals for the variants. The darkness represents the level of U1-gene expression.

As a core component of the spliceosome during the early assembly stage for intron recognition, U1 snRNA and its assembled U1 snRNP are essential for pre-mRNA splicing, which is confirmed by the lethality of *21D* & *95Ca* double-deletion strain. However, deletion of singular U1-gene does not result in significant changes in pre-mRNA splicing, regarding fewer changed AS events in the deletion strains compared to strains with mutations in other splicing factors. In addition, no annotated splicing factors (including spliceosomal components and splicing regulators) were found in the DEGs in most of the U1 deletion strains, and a small number of splicing factors were found in the APA-genes, suggesting that the detected DASs are likely indirect effects of gene expression or APA changes and the function of each U1-gene in pre-mRNA splicing could be compensated by the other copies of U1-genes (Fig 8).

Large amounts of genes’ expression and 3’-end formation are seriously affected in the singular U1-gene deletion strains, in which most are down-regulated genes or activation of the proximal sites for 3’-end polyadenylation. These results suggest that each U1-gene could bind to the 5’ splice sites of specific nascent pre-mRNAs and affect their transcription and 3’-end formation in a way that is coupled with transcription (Fig 8). Enrichment of genes with 1–2 introns or shorter introns, or genes in shorter length among the DEGs suggests that when a U1-gene was deleted, the transcription would be easily decreased or paused at its early transcribed region containing U1-binding sites, which is consistent with a recently published work that U1 snRNP increases RNA Pol II elongation rate to enable synthesis of long genes [63]. This study is also consistent with the previous finding that knockdown of the total U1 results in premature cleavage and polyadenylation in numerous pre-mRNAs at cryptic sites, frequently in introns near the start of the transcript [51].

In addition, we find that 20.6–25.8% of the down-regulated DEGs and 19.5–30.5% of the up-regulated DEGs caused by the U1-gene KOs are intronless genes, slightly different from its ratio in the fly genome (28.8%). However, only 1.5–8.8% of APAs caused by the U1-KOs are from intronless genes, significantly lower than the ratio in the genome (S9A Fig), suggesting that the APAs caused by single U1-KOs are more connected with intron-containing genes (or with splicing), but the DEGs caused by single U1-KOs are not specifically connected with splicing. We also retrieved the intronless genes with APA events, in which there are no more than five genes whose expression is significantly changed in each of the U1-deletion strains (S9B Fig). At present, we do not know how exactly the expression of intronless genes was regulated by U1 snRNA. The down-regulated intronless genes would have potential U1 binding sites, which are regulated by the transcriptional coupling; the up-regulated intronless genes would be the results of indirect effects.

### Relationship between the singular U1-gene regulated DEGs, DASs and APAs

As mentioned above, there are large amounts of differentially-expressed genes (DEGs) and alternative polyadenylation events (APAs) but lower levels of AS changes (DASs) in each of the U1-KO strains. We compared each two of the three sets of data and found that their overlapping events are at very low levels (S9C and S9E Fig), suggesting that the U1-gene KO caused changes in gene expression, alternative splicing, and alternative polyadenylation would be through relatively independent mechanisms.

Most of the DEGs in the U1-KO strains are down-regulated, which is consistent with previous reports that the U1 snRNP is coupled with Pol II CTD and enhances the transcription [63,64]. To validate this coupling mechanism, we performed Pol II ChIP- seq twice using the 3^rd^ larva samples and a monoclonal 4H8 antibody from two companies, Santa Cruz Biotechnology (sc-44701) and Abcam (ab5408). This antibody recognizes both the phosphorylated and unphosphorylated forms of the largest subunit of RNA pol II. Unfortunately, we did not obtain high-resolution signals due to the technical challenge in flies. We only identified around one hundred peaks in each of the U1-deletion samples. Fortunately, among those peaks, we can see changes in Pol II-bindings on about 10 DEGs in each sample. Five representative down-regulated DEGs (one from each KO strain) showed increased Pol II binding (S10 Fig), suggesting that transcription of these DEGs was affected when a specific U1-gene was KO. We also observed up-regulated genes in the *Drosophila* mutants, which could be caused by indirect effects. It will be interesting to address the tissue-specific regulations of gene expression, alternative splicing and polyadenylation when a specific U1-gene was KO.

### Similarities and differences between the five U1 genes

By the alignment of the upstream sequences, we find that three U1-genes, *21D*, *82Eb*, and *95Ca*, display closer similarity than the other two U1-genes (S2 Fig). This similarity is consistent with the analysis of transcription factors binding based on online ChIP-seq data (S4A Fig), and also consistent with the PCA analysis of expression-changed genes (S5B Fig). Taken together, we speculate that transcription of these three U1-genes could be regulated by some common factors.

We also observe many differences and puzzles between the five U1-genes. Firstly, the *82Eb* deletion strain exhibits the most amounts of DEGs and APA events, which is consistent with the extent of its seriously defective phenotypes. However, it remains unclear why deletion of the ubiquitously-expressed *21D* does not show the most changes in DEGs and 3’-end formation. Secondly, the SmD2 pull-down assay indicates that the three variants of U1 snRNA have different abilities in snRNP assembly. Thirdly, analysis of the overall U1 levels in the singular U1-gene deletion strains suggests that it is either the tissue-specific expression or the nucleotide differences of U1s that contribute to the changes of transcription, alternative splicing, and polyadenylation. One possibility to answer these puzzles could be the U1 variants with decreased Sm protein binding ability, especially the *82Eb*, might form a different U1 snRNP that has a tighter coupling with transcription and polyadenylation.

## Materials and methods

### Fly strains and culture

The wild-type (*WT*) *Drosophila melanogaster* used in this study is a *w1118* isogenic strain (BDSC 5905). Five U1-driven GFP transgenic strains were constructed using the PhiC31 integrase system [65]. Briefly, for each strain, a transgenic pBID plasmid containing the upstream 1.5 kb of U1 gene, Kozac sequence, CDS of GFP, and the downstream 500 bp of U1 gene (Fig 1B) was injected into embryos of strain with attP site (*y* [*1*]*M*[*vas-int*. *Dm*] *ZH-2A w* [*]; *P[CaryP]attP40*), and incorporated in chromosome 2L. Five U1-gene deletion strains were constructed using CRISPR/Cas9-mediated gene editing [61]. Briefly, plasmids expressing guide RNAs (sgRNA) that specifically target the upstream and downstream sequences of each U1 snRNA locus were constructed (Fig 3A), and then micro-injected in embryos of *nanos-Cas9* strain at the Core Facility of Drosophila Resource and Technology, CEMCS, CAS. All the acquired strains were screened by genomic PCR using specific primers (listed in S5 Table), which were further validated by Sanger sequencing. The final obtained lines were then crossed with the WT strain (BDSC 5905) for at least five generations to eliminate potential off-target events. The *WT*, mutant, and tool strains were maintained and cultured on standard cornmeal agar medium, and listed in S6 Table.

### RT-PCR and qPCR

Total RNA was extracted with TRIzol Reagent (Invitrogen) according to the manufacturer’s protocol. After treatment with DNase I (Takara), reverse transcription (RT) was carried out using the RevertAid First Strand cDNA Synthesis Kit (Thermo) with oligo(dT) and random hexamers for the synthesis of cDNA, which was then amplified by the PrimeSTAR Max DNA Polymerase (Takara). Quantitative PCR (qPCR) was performed using the SYBR Green Realtime PCR Master Mix (Toyobo) and a BioRad real-time PCR system. Relative expression of genes was then normalized by the *gapdh* mRNA.

### Confocal microscope imaging

Dissected tissues from the *WT* and GFP transgenic flies, including female and male reproductive systems from adults and the salivary gland from the 3^rd^ instar larvae, were placed in the fixing solution (Beyotime) and fixed at 4°C. Washed with 1×PBS, the tissues were then treated with membrane-breaking solution (Beyotime) and PBS washed again. DAPI (1:2000; Sigma) was used for staining the nuclei. Images were acquired using Carl Zeiss LSM880 confocal microscope.

### Developmental assays of *Drosophila*

The developmental assays were all performed under standard conditions. For hatching rates, 100 laid eggs from each mating were collected and passed to new vials, the hatched larvae were then counted. For pupation rates, 100 of the 1^st^ instar larvae were collected and cultured, and the pupae were counted 5 days later. Similarly, the number of adults that eclosed from 100 pupae of each strain was used to calculate the pupation rates. Adult lifespans were measured as described [66]. Briefly, 100 virgins (equally in genders) were maintained at a density of 25 per vial with standard food. Flies were transferred to new vials every 2–3 days and the dead flies were counted. All the above tests were performed in triplets, the statistics were analyzed by GraphPad Prism 8 (San Diego), and the significant differences were determined according to *t-*tests.

### Larva locomotion and adult climbing

Locomotion of the 3^rd^ instar larvae was detected with slight modification as described [61]. Eight of the larvae from each strain were randomly selected and placed in a 6-cm plate, and 0.5 ml of 1×PBS were added to keep the larval body wet. The larval movement was recorded in a 1-min video, and the video was imported into Photoshop to draw the movement track.

Climbing of adults (40 for each strain) was performed as described [42]. Briefly, the fruit flies were placed at the bottom of the tube, and their climbing distances were measured 1 min after shaking. The distance was divided into three stages: low, 0–6 cm height; medium, 6–12 cm height; high, 12–18 cm height. All the above tests were performed in triplets, the statistics were analyzed by GraphPad Prism 8 (San Diego), and the significant differences were determined according to *t-*tests.

### RNA-seq and bioinformatics

Total RNA from the 3^rd^ instar larvae of the WT and the five U1-gene deletion strains were isolated by TRIzol (Invitrogen) and treated with RNase-free DNase I (Takara). Construction of cDNA libraries and sequencing were performed using Illumina HiseqXten-PE150 by Novogene. Raw reads from RNA-seq were quality filtered and trimmed by *Trimmomatic* [67] and were then mapped to the *D*. *melanogaster* genome (*dm6*) by *HISAT2* [68] and counted by *featureCounts* [69].

Analyses of differentially expressed genes (DEGs) were performed by *DEseq2* [70], and genes with fold-changes > 2 and FDR < 0.05 were screened as significant. The Gene Ontology (GO) enrichment is analyzed by *clusterProfiler* [71]. Differentially spliced (DS) events were analyzed by *rMATS* [72], in which the significant DS events were screened by conditions of |*ΔPSI*| > 0.05 and FDR < 0.05. Enrichment of DEGs was performed according to intron numbers of genes and length of pre-mRNAs using *R*, and the *ggplot2* package was used for visualization. *Trans*-splicing levels of each *mod(mdg4)* and *lola* isoforms were analyzed through mapping of their specific exon-exon junction reads by *Hisat2* [68], which were normalized by sequencing depth and their common exon reads. Alternative 3’-end polyadenylation events were investigated by *DaPars* [73], and events with |*ΔPDUI*| > 0.25 and FDR < 0.05 were screened as significant.

### In vitro transcription of U1 snRNA variants and pull-down assay

Canonical and variants of U1 snRNA were in vitro transcribed using the MEGAshortscript High Yield Transcription Kit (Invitrogen), which were attached with a single biotinylated nucleotide to the 3’-terminus using the RNA 3’-end Biotinylation Kit (Pierce). One hundred pmoles of the biotinylated U1 snRNA were added into the lysate from 1×10^7^ S2 cells, and the pull-down assay was performed as described protocol in the Magnetic RNA-Protein Pull-Down Kit (Pierce). The protein-associated beads were then boiled in 1 x SDS buffer and analyzed by western blotting using an anti-SmD2 antibody (Abcam).

## Supporting information

S1 Fig Multiple copies with variations of spliceosomal snRNA genes exist in high eukaryotes. (**A**) List of copy numbers of the major spliceosomal snRNA genes in model systems according to annotations in the NCBI database. (**B**) Sequences and secondary structure of U1 snRNAs from the five Drosophila U1-genes. Variations and positions in the two U1-genes (*82Eb* and *95Cc*) are shown in red. The predicted secondary structure of U1 snRNA is adopted [27]. Rectangles indicate the recognition motif to the 5’ splice site (blue) and Sm protein binding site (red).
(TIF)

S2 Fig Alignment of the upstream 1Kb sequences of the *Drosophila* five U1-genes. The alignment was performed by Jalview. The three U1 genes (*21D*, *82Eb*, and *95Ca*) have closer sequence similarities at their promoter regions than the other two U1 genes (*95Cb* and *95Cc*).
(TIF)

S3 Fig Fluorescent signals in the transgenic U1-gene-driven GFP strains. GFP signals were detected for both gender adults (**A**) and female ovaries (**B**) by stereoscope imaging.
(TIF)

S4 Fig Binding of transcription factors on *Drosophila* U1 genes through analyses of online ChIP-seq data and viabilities of double U1-genes deleted *Drosophila* strains. (**A**) Forty-five transcription factors (TFs) bind on the *Drosophila* U1 genes after analyses of the online data from ChIP-Atlas. *Drosophila* TFs are defined by AnimalTFDB. Blue rectangles, TFs have binding peaks on the U1-gene in any of the *Drosophila* samples from 11 tissues or 5 developmental stages. (**B**) In the salivary gland, the *95Ca* gene is strongly co-immunoprecipitated only by Trl, which is a GAGA transcription factor involved in chromatin modification [57]. (**C**) Cross table of homozygotic double U1-gene deletions from the five singular U1-deletion strains. n.a., crosses between the three 95C genes were not successful due to their short physical distances. (**D**) Cross table of heterozygotic and homozygotic double-deletions between the *21D* and *95Ca* genes.
(TIF)

S5 Fig Overlapping and GO analyses of the differentially expressed genes in the five U1-deletion strains. (**A**) Overlapping of the DEGs in the five U1-deletion strains. (**B**) Principal component analysis (PCA) of the five U1-deletion strains. (**C**) GO analyses of the down- and up-regulated DEGs in the five U1-deletion strains. (D) GO analyses of the commonly down-regulated DEGs. (E) Most of the commonly down-regulated DEGs are highly expressed in the midgut based on analysis of expression data from FlyBase.
(TIF)

S6 Fig Alternative splicing changes and distribution of the differentially-expressed-genes that are classified by their containing introns in the five U1-deletion strains. (**A**) Five types of alternative splicing events presented with changed PSI in the U1-KO samples. (**B**) Overlapping of the AS events between the five U1-KO samples. (**C**) Density distribution of DEGs that are classified by numbers of containing introns. DEGs are grouped as down-regulated genes (brown) and up-regulated genes (blue). Genes transcribed in the sequenced WT strains are analyzed as a control.
(TIF)

S7 Fig Enrichment of differentially-expressed genes that are classified by gene and intron length and GC contents, and classification of APA events in the five U1-deletion strains. Enrichment analyses are performed for all the DEGs and the down-regulated DEGs in the U1-gene deletion strains according to their gene length (**A**), intron length (**B**), and GC contents (**C**). (**D**) APA events are classified into two groups: in 3’-UTR regions and in the terminal exon/intron regions.
(TIF)

S8 Fig Examples of APA events in the U1-gene deletion strains. (**A**) Five APA events that favor selection of proximal sites in the U1-deletion strains. (**B**) Ten APA events that favor selection of distal sites in the U1-deletion strains. Brown IGVs, DPDUI < -0.25, favor proximal sites; red IGVs, DPDUI > 0.25, favor distal sites. (**C**) and (**D**) Validation of the APA events by RT-qPCR. The amplification of the long 3’-UTR region represents the selection of distal site, and the amplification of the common region of all the 3’-UTR isoforms represents both selections (distal + proximal). The relative distal selection is presented by the ratio of distal-to-common in each strain, which is normalized by the value in WT. All data are shown as means ± SEM. *, p < 0.05; **, p < 0.01; ***, p < 0.001.
(TIF)

S9 Fig Changes of the intronless genes and comparison of three kinds of events in the five U1-gene KO strains. (**A**) The ratio of intronless genes in the APA events and DEGs. Annotated fly genes are used as control. (**B**) Overlapping of intronless genes between the APA events and DEGs. (**C**) Overlapping of the DEGs and the APA events in the U1-KO strains. (**D**) Overlapping of the DEGs and the DAS events in the U1-KO strains. (**E**) Overlapping of the DAS events and the APA events in the U1-KO strains. The overlapping number is shown before the slash in each of the comparisons.
(TIF)

S10 Fig Examples of the Pol II binding on the expression-changed genes in the five U1-gene KO strains. Pol II ChIP-seq was performed using the monoclonal antibody 4H8. Blue bars, the identified Pol II binding peaks that are enhanced in the U1-KO samples. Both ChIP-seq and RNA-seq data are presented for each gene.
(TIF)

S1 Table Reads statistics of RNA-seq in this study. (DOCX)

S2 Table Differentially expressed genes in the U1-gene deletion strains. (XLSX)

S3 Table Differentially alternatively spliced events in the U1-gene deletion strains. (XLSX)

S4 Table Alternative polyadenylation events in the U1-gene deletion strains. (XLSX)

S5 Table List of primers used in this study. (DOCX)

S6 Table List of *Drosophila* strains used in this study. (DOCX)
