# Supplementary figures and images for: Deletions of singular U1 snRNA gene significantly interfere with transcription and 3’-end mRNA formation
Source: PLoS Genet. 2023 Nov 2;19(11):e1011021. doi: 10.1371/journal.pgen.1011021 (PMC10645366; doi:10.1371/journal.pgen.1011021)

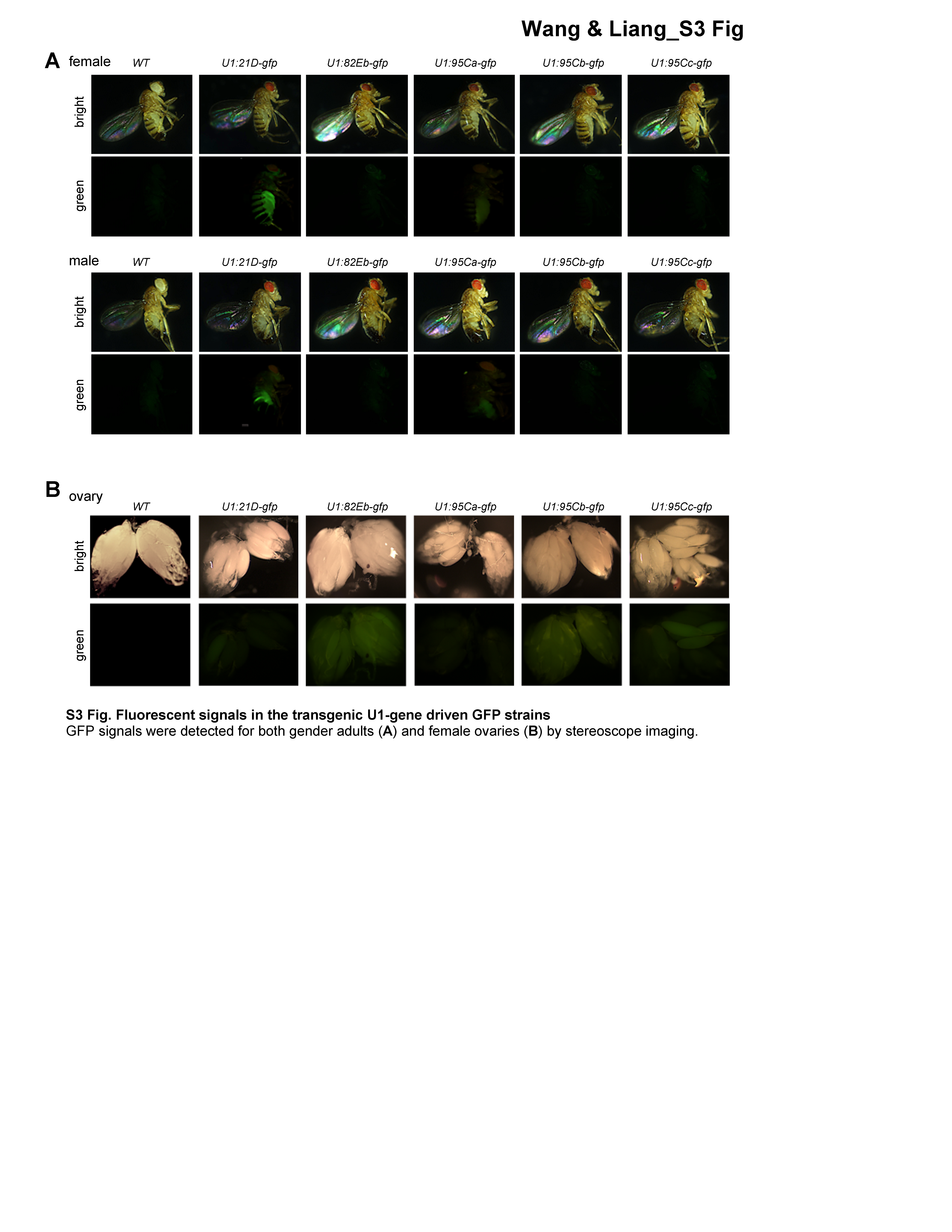

Supplement: S3 Fig — GFP signals were detected for both gender adults (A) and female ovaries (B) by stereoscope imaging. (TIF) [file pgen.1011021.s003.tif]
