# Supplementary material for: Deletions of singular U1 snRNA gene significantly interfere with transcription and 3’-end mRNA formation
Source: PLoS Genet. 2023 Nov 2;19(11):e1011021. doi: 10.1371/journal.pgen.1011021 (PMC10645366; doi:10.1371/journal.pgen.1011021)
Supplement: S1 Table — (DOCX) [file pgen.1011021.s011.docx]

**Wang & Liang S1 Table**

**S1 Table. Reads statistics of RNA-seq in this study**

| **Samples** | | **Raw reads** | **Clean reads** | **Mapped reads** | **Total mapped%** |
| --- | --- | --- | --- | --- | --- |
|  | *WT_1* | 35,370,101 | 34,286,495 | 30,813,273 | 89.87% |
|  | *WT_2* | 26,701,169 | 25,364,206 | 20,570,371 | 81.10% |
|  | *WT_3* | 35,270,854 | 34,168,084 | 31,465,389 | 92.09% |
|  | *21D^∆/∆^_1* | 32,837,365 | 32,332,054 | 28,177,385 | 87.15% |
|  | *21D^∆/∆^_2* | 29,971,207 | 28,770,646 | 23,266,821 | 80.87% |
|  | *21D^∆/∆^_3* | 34,054,239 | 32,845,305 | 30,092,868 | 91.62% |
|  | *82Eb^∆/∆^_1* | 33,689,822 | 32,774,812 | 29,749,697 | 90.77% |
|  | *82Eb^∆/∆^_2* | 26,693,344 | 26,010,593 | 22,161,025 | 85.20% |
|  | *82Eb^∆/∆^_3* | 29,063,306 | 28,069,648 | 25,366,541 | 90.37% |
|  | *95Ca^∆/∆^_1* | 36,029,380 | 34,813,319 | 29,479,919 | 84.68% |
|  | *95Ca^∆/∆^_2* | 36,068,831 | 33,927,915 | 31,376,536 | 92.48% |
|  | *95Ca^∆/∆^_3* | 26,991,053 | 26,077,996 | 23,629,272 | 90.61% |
|  | *95Ca^∆/∆^_1* | 30,988,150 | 30,388,675 | 27,206,981 | 89.53% |
|  | *95Ca^∆/∆^_2* | 31,254,111 | 30,631,467 | 26,925,059 | 87.90% |
|  | *95Ca^∆/∆^_3* | 24,461,965 | 23,501,676 | 21,753,151 | 92.56% |
|  | *95Ca^∆/∆^_1* | 30,461,511 | 29,915,465 | 26,418,347 | 88.31% |
|  | *95Ca^∆/∆^_2* | 26,625,406 | 25,842,951 | 22,984,721 | 88.94% |
|  | *95Ca^∆/∆^_3* | 28,268,342 | 27,378,353 | 24,522,791 | 89.57% |

Total RNAs were isolated from the *Drosophila* 3^rd^ instar larvae and then performed Illumina mRNA-seq.
