# Supplementary material for: Deletions of singular U1 snRNA gene significantly interfere with transcription and 3’-end mRNA formation
Source: PLoS Genet. 2023 Nov 2;19(11):e1011021. doi: 10.1371/journal.pgen.1011021 (PMC10645366; doi:10.1371/journal.pgen.1011021)
Supplement: S5 Table — (DOCX) [file pgen.1011021.s015.docx]

**Wang & Liang S5 Table**

**S5 Table. Primers used in this study**

| **Primers** | **Sequence** | **Notes** |
| --- | --- | --- |
| WM001 | 5'GAAGATACCACTTCGCGATC | U6a-sgRNA 5’up-369 F |
| WM002 | 5'ATCTCGGCATCATCCAGAGG | U6a-sgRNA 3’down-155 R |
| WM003 | 5'TTCGCTTTCCTCAGCTCAGGGAAT | Dm U1:21D-upF |
| WM004 | 5'AAACATTCCCTGAGCTGAGGAAAG | Dm U1:21D-upR |
| WM005 | 5'TTCGAAGGAAGGAACGTTCCTCAT | Dm U1:21D-downF |
| WM006 | 5'AAACATGAGGAACGTTCCTTCCTT | Dm U1:21D-downR |
| WM007 | 5'TTCGAGGGCGCAACTAGAACTAGT | Dm U1:82Eb-upF |
| WM008 | 5'AAACACTAGTTCTAGTTGCGCCCT | Dm U1:82Eb-upR |
| WM009 | 5'TTCGCTGTTTTAATCAGGCGTTTA | Dm U1:82Eb-upF |
| WM010 | 5'AAACTAAACGCCTGATTAAAACAG | Dm U1:82Eb-upR |
| WM011 | 5'TTCGTTACCTCCTATCGACTCAGC | Dm U1:95Ca-upF |
| WM012 | 5'AAACGCTGAGTCGATAGGAGGTAA | Dm U1:95Ca-upR |
| WM013 | 5'TTCGGCAAAATGTTATGAACAACG | Dm U1:95Ca-downF |
| WM014 | 5'AAACCGTTGTTCATAACATTTTGC | Dm U1:95Ca-downR |
| WM015 | 5'TTCGAGTGCAATGTAGGCACTGCT | Dm U1:95Cb-upF |
| WM016 | 5'AAACAGCAGTGCCTACATTGCACT | Dm U1:95Cb-upR |
| WM017 | 5'TTCGCGAGGTAGTCTCGCTAGCTT | Dm U1:95Cb-downF |
| WM018 | 5'AAACAAGCTAGCGAGACTACCTCG | Dm U1:95Cb-downR |
| WM019 | 5'TTCGTCTAGGATATCGGTCATGAC | Dm U1:95Cc-upF |
| WM020 | 5'AAACGTCATGACCGATATCCTAGA | Dm U1:95Cc-upR |
| WM021 | 5'TTCGGCGCAAGCTAGCTCTGTTAG | Dm U1:95Cc-downF |
| WM022 | 5'AAACCTAACAGAGCTAGCTTGCGC | Dm U1:95Cc-downR |
| WM023 | 5'TTAActcgagTAATTGCGTAGAAAACGCACTC | Dm XhoI-U1-21D up1500F |
| WM024 | 5'AGCTCCTCGCCCTTGCTCACCATGTTGGCTTTCCTCAGCTCAGGGAAT | Dm U1-21D up kozac *gfp*-R |
| WM025 | 5'ATGGTGAGCAAGGGCGAGGAG | *gfp*-F |
| WM026 | 5'TTACTTGTACAGCTCGTCCATG | *gfp*-R |
| WM027 | 5'TTAActcgagGATTGCTACTATATAATTACTGCAT | Dm XhoI-U1-95Ca up1500F |
| WM028 | 5'AGCTCCTCGCCCTTGCTCACCATGTTGGCTTTTCGATGCTCGGCAG | Dm U1-95Ca up kozac *gfp*-R |
| WM029 | 5'TGGACGAGCTGTACAAGTAACATCTGCAATAAATTTTAAAAGTATC | 3'*gfp*-U1-21D terminator F |
| WM030 | 5'CCTTCGACCGTGCGATCGA | Dm U1-21D down500R |
| WM031 | 5'TGGACGAGCTGTACAAGTAACATTTATAATAAATATTCAAACTATTAAGG | 3'*gfp*-U1-95Ca terminator F |
| WM032 | 5' GCATGAGTCGCTGGCTAAAAGG | Dm U1-95Ca down 540R |
| WM033 | 5' GACCACGCGTAACTCTTATCA | attP40-up540F |
| WM034 | 5' GTTGAAAGATCAATGGAGTACT | attP40-up350F |
| WM035 | 5' TCTCACGCAGTTAGGTAAGGA | attP40-down300R |
| WM036 | 5' GCAAATACACACGCTTCTTGG | attP40-down 400R |
| WM037 | 5' GTTGTCAGGTTAACCATATTCC | *U1:21D*-F |
| WM038 | 5' GCAGATGTTCTTCATGGTGGTG | *U1:21D*-R |
| WM039 | 5' TGACTGCAGTCAAACACACTAG | *U1:82Eb*-F |
| WM040 | 5' GTAGTCGAGGACAACTTCCACA | *U1:82Eb*-R |
| WM041 | 5' GCATTCCGGAGTTGCAAGTAGAT | *U1:95Ca*-F |
| WM042 | 5' TATTCATGTCTTTGGTTGCCAA | *U1:95Ca*-R |
| WM043 | 5' GATGACGACGTCAAAGTCGAAGA | *U1:95Cb*-F |
| WM044 | 5' CTCCAATCCGCTCGACAAGTGC | *U1:95Cb*-R |
| WM045 | 5' GTTGCGCTGATTTCCAGCTGAG | *U1:95Cc*-F |
| WM046 | 5' TGGGAATGTTCAGGGATTTCCA | *U1:95Cc*-R |
| WM047 | 5' GCACAAGCTGGAGTACAACTA | *gfp* test-R |
| WM048 | 5' TGTTGTGGCGGATCTTGAA | *gfp* test-F |
| WM049 | 5' CCTGGTGGTGAACGGCCAGAAGATC | *gapdh*-F |
| WM050 | 5' CCAGGTTAACGCCGCACACGAAC | *gapdh*-R |
| WM051 | 5' GGCGTAGAGGTTAACCGTGAT | U1-F |
| WM052 | 5' CGCACGAGTTATTCACATTAGG | U1-R |
| WM053 | 5' ATCGCTTCTCGGCCTTATGGCTAAG | U2-F |
| WM054 | 5' GTTGGGCCGAAATCCCGGCGGTACTG | U2-R |
| WM055 | 5' GCGCAGTGGCAATACCGTAACC | U4-F |
| WM056 | 5' TGCCGTAGTGGACGGTATTTCAC | U4-R |
| WM057 | 5' ACTCTGGTTTCTCTTCAATTGTC | U5-F |
| WM058 | 5' AGACTCATTAGAGTGTTCCTCTC | U5-R |
| WM059 | 5' GTTCTTGCTTCGGCAGAACATATAC | U6-F |
| WM060 | 5' TGTGGAACGCTTCACGATTTTGCG | U6-R |
| WM061 | 5' CGTTTCCGATCACGAAAC | U11-F |
| WM062 | 5' CGCAGGGAGATCCGGGAAT | U11-R |
| WM063 | 5' CTAATGAGTAAGGAAAAC | U12-F |
| WM064 | 5' AAGTAGGCGCCGGTTCGCT | U12-R |
| WM065 | 5' GCCTTGCTGGAGCTGGAAACG | FBgn0030776-F |
| WM066 | 5' GCATCACCGGAACAGAGTCCGG | FBgn0030776-R |
| WM067 | 5' CATAGCTACCTTCTCCCATGGC | FBgn0031522-F |
| WM068 | 5' CGCAATGAAGAAAAGGACCATC | FBgn0031522-R |
| WM069 | 5' CGGGATTGGCCATTCCGCTGCC | FBgn0032304-F |
| WM070 | 5' CAACTTGCACTTGGAACCCAC | FBgn0032304-R |
| WM071 | 5' CTCTTCCTCATCGAGCTATCCTGC | FBgn0029848-F |
| WM072 | 5' CCAAAGGTTACGCCAAAGTCTGTC | FBgn0029848-R |
| WM073 | 5' GACAGCCGCCCCTGGTGGGAACG | FBgn0000078-F |
| WM074 | 5' GACCACCTTGTCCTGCACGTAG | FBgn0000078-R |
| WM075 | 5' GCCACCGAGTGGTGGGAGAGTGG | FBgn0002570-F |
| WM076 | 5' GAACCATTCGTTTTCGGTACTTGAG | FBgn0002570-R |
| WM077 | 5' GCCAAGCTCTCCGACAAGCTGG | FBgn0031249-F |
| WM078 | 5' GTAGGGCCCAACGCCACCGGTG | FBgn0031249-R |
| WM079 | 5' GGGGCCCAGGCTGTGGACTGG | FBgn0035670-F |
| WM080 | 5' GGTGCGATCGTGGGCGCCCAGG | FBgn0035670-R |
| WM081 | 5' CGCCTTCGACGAGAAGGTCTTC | FBgn0031653-F |
| WM082 | 5' CGTTGCCAACGGTGTGGGTGAAC | FBgn0031653-R |
| WM083 | 5' GAGCACATCAAGGCCACGTCTCCG | FBgn0023495-F |
| WM084 | 5' GAACGGCACCAGGTAGTCCAGCG | FBgn0023495-R |
| WM085 | 5' GGCACCTGCGACCAGTACATTCAG | FBgn0260393-F |
| WM086 | 5' GTACAGGCAGGACTGAGAGCG | FBgn0260393-R |
| WM087 | 5' AGCATCAGTTGTCAACGGCTAA | FBgn0040736-F |
| WM088 | 5' CGTTGGCCAGAGCCAGCAGACCC | FBgn0040736-R |
| WM089 | 5' CACTGTACACACCGGAGTGGGTGAC | FBgn0002939-F |
| WM090 | 5' CGGCGTTATCTCGTACTTCCAACC | FBgn0002939-R |
| WM091 | 5' CTTGGCTGGACTACGATCTGGGCTTC | FBgn0034582-F |
| WM092 | 5' CGAATCCATGGTTGCGCAGCAGGTTC | FBgn0034582-R |
| WM093 | 5' GTTGGGGTAGTGGACATCCTTGG | FBgn0262722-F |
| WM094 | 5' CGGTATGTGCTTTATTGTTGAGTT | FBgn0262722-R |
| WM095 | 5' CTCTG GGCTGCATCA CTTCCAGC | FBgn0038717-F |
| WM096 | 5' AACTTGGCGACACCTCGAAGAC | FBgn0038717-R |
| WM097 | 5' CGCACACGACAATTGGTGACCATC | FBgn0039872-F |
| WM098 | 5' CGCTTCCCTGGACCACATCTGTCC | FBgn0039872-R |
| WM099 | 5' GTTGAGCGCGTAATGCACGTTATG | FBgn0259164-F |
| WM100 | 5' GGCGACGTCTACGGCTACTGCAG | FBgn0259164-R |
| WM101 | 5' CAACATGAAGTTCTTCGTAAGTGGC | FBgn0086519-F |
| WM102 | 5' CTCGGCACGGAGAACATTGGCATCTTC | FBgn0086519-R |
| WM103 | 5' CGGCCTGCAGGAGGTGGAGCAGCA | FBgn0003082-F |
| WM104 | 5' CGCAGCGGAGCAAAGTCACAGACTAC | FBgn0003082-R |
| WM105 | 5' CTGCTGTCTTTGCTCTTGATGATCC | FBgn0052198-F |
| WM106 | 5' CAGTGGTGGCAGTAGAGGCATCTGGC | FBgn0052198-R |
| WM107 | 5' CGACGTCGTGATGCAGCTGCTCTAC | FBgn0035138-F |
| WM108 | 5' CGGTCAGCACGAGTGAAACGGCC | FBgn0035138-R |
| WM109 | 5' GTGAGCAGCGTTATCTGTCCGGAG | FBgn0039805-F |
| WM110 | 5' GATAGCTGTAGCTGCCATGACGTGT | FBgn0039805-R |
| WM111 | 5' CGCAGCTCCTGGTGTCCTGTGCCC | FBgn0043577-F |
| WM112 | 5' CTTTCTGCGCATATGATCGGCTC | FBgn0043577-R |
| WM113 | 5' GATTGTTACCGCGATACTGTCCATG | *mod(mdg4)*-D-R |
| WM114 | 5' CAATCCACTGGCCACGCGGAATATC | *mod(mdg4)*-K-R |
| WM115 | 5' CTTTTTGAATCGGTGCTATATGTC | *mod(mdg4)*-S-R |
| WM116 | 5' GCTCACGGTAGCAGCACTGAAACAC | *mod(mdg4)*-T-R |
| WM117 | 5' GCAACCACCTTGGCAATCGTCTC | *mod(mdg4)*-V-R |
| WM118 | 5' GTAGGTTGTGGTATCGTGCTCGTTG | *mod(mdg4)*-W-R |
| WM119 | 5' TGAATCATCGTTTGTCGACACCAG | *mod(mdg4)*-common-F |
| WM120 | 5' CAGTCGTCCAGTTCGAATTCATC | *lola*-AA-R |
| WM121 | 5' CTCATCCCGCTGCTTGCCATTGAG | *lola*-G-R |
| WM122 | 5' CTGGCGGAAGTGGCCATCGCCGTAG | *lola*-Q-R |
| WM123 | 5' GATGCACAGCAACGGGATCCCCAAG | *lola*-common-F |
| ZZZ01 | 5' GTTGCCAGCTACGACCGTGTCCGTG | *CG10119*-common-F |
| ZZZ02 | 5' GCTCCCGCCTAGAAGAGCAGGGAG | *CG10119*-common-R |
| ZZZ03 | 5' GCTTGGATCCTGAGGCCCAGC | *CG10119*-distal-F |
| ZZZ04 | 5' GCTTGATATGCAGAACAGATCTGTG | *CG10119*-distal-R |
| ZZZ05 | 5' GATGCTGCTCAGGGCAAGTA | *CG6058*-common-F |
| ZZZ06 | 5' GTGTAGCCAAGGTCCAACCA | *CG6058*-common-R |
| ZZZ07 | 5' TCGAGAGCTAAGCAAAGCAA | *CG6058*-distal-F |
| ZZZ08 | 5' TCCTTTCTTCTGCACTCATCA | *CG6058*-distal-R |
| ZZZ09 | 5' CTACATAGCCAGTCCCGCAG | *CG11089*-common-F |
| ZZZ10 | 5' TGGGCCATGATGATACCGTG | *CG11089*-common-R |
| ZZZ11 | 5' ACACCCTGTTAATCAAACCCCA | *CG11089*-distal-F |
| ZZZ12 | 5' AAAGGCAAGTGGTTTTATTGAGCTT | *CG11089*-distal-R |
| ZZZ13 | 5' CGATGCACTGCTTGTCGAAG | *CG1600*-common-F |
| ZZZ14 | 5' ATGGCGATGATCTCCTGCTG | *CG1600*-common-R |
| ZZZ15 | 5' TTTGGACTGCAGACGAACTC | *CG1600*-distal-F |
| ZZZ16 | 5' TATGGCGATGTGATGGGTGA | *CG1600*-distal-R |
| ZZZ17 | 5' ACCACTGCTGAACATGCCAA | *Tango1*-common-F |
| ZZZ18 | 5' TCCAGTTGGAGGCAGGAATG | *Tango1*-common-R |
| ZZZ19 | 5' CATTCCTGCCTCCAACTGGA | *Tango1*-distal-F |
| ZZZ20 | 5' CGTCGACGACGGTCCATG | *Tango1*-distal-R |
| ZZZ21 | 5' AGATCAACACCATCCTGAAGGC | *CG4087*-common-F |
| ZZZ22 | 5' GGTGATCAGGTCCTTGACGTT | *CG4087*-common-R |
| ZZZ23 | 5' TTCCTTTCACAACGGTGCAG | *CG4087*-distal-F |
| ZZZ24 | 5' TCAACCCGGCTGACCAAATTA | *CG4087*-distal-R |
| ZZZ25 | 5' GGAATTGTTCATGTGCTCCGT | *CG7073*-common-F |
| ZZZ26 | 5' GCTGGTGGTGGTTGCTAATG | *CG7073*-common-R |
| ZZZ27 | 5' ACCACCAGCACCATATTTTCA | *CG7073*-distal-F |
| ZZZ28 | 5' TCTGACGGTTTGAATTGGGC | *CG7073*-distal-R |
| ZZZ29 | 5' CGTTAAGCGCCAAGTTCTGA | *CG7033*-common-F |
| ZZZ30 | 5' CGTGTAAGGTTAGCGATGATGT | *CG7033*-common-R |
| ZZZ31 | 5' TCGCTAACCTTACACGTTCTG | *CG7033*-distal-F |
| ZZZ32 | 5' AGGATTACACGACAGGGACA | *CG7033*-distal-R |
| ZZZ33 | 5' CCGGTTGTTGTTAAGGGAAA | *CG7748*-common-F |
| ZZZ34 | 5' CGATTAAAGCCCTCAACATGA | *CG7748*-common-R |
| ZZZ35 | 5' CACCGCATCCTCATGTTGAG | *CG7748*-distal-F |
| ZZZ36 | 5' ACGAAAGCAGCAACTCGATAA | *CG7748*-distal-R |
| ZZZ37 | 5' GAGGTGATGCGCATGTGTAA | *CG12963*-common-F |
| ZZZ38 | 5' CGGACGAGGAATGTGGATCT | *CG12963*-common-R |
| ZZZ39 | 5' TCGCAATGGAACCGATGGAG | *CG12963*-distal-F |
| ZZZ40 | 5' CGAATGCTTCAGTTCCAATTTATCC | *CG12963*-distal-R |
| ZZZ41 | 5' ATGAGCCGCCCACTAAAGTT | *CG12592*-common-F |
| ZZZ42 | 5' GCTTCCTTGACATCCCGGAG | *CG12592*-common-R |
| ZZZ43 | 5' GCATTTCCCCGATTGAATTTG | *CG12592*-distal-F |
| ZZZ44 | 5' CTTGCTAGCGTTAAGTGTGGT | *CG12592*-distal-R |
| ZZZ45 | 5' GCATGATCCAGTCGACGATG | *Fbp2*-common-F |
| ZZZ46 | 5' GGTTCCAGGCCATAGACAGA | *Fbp2*-common-R |
| ZZZ47 | 5' AGCCATGCAAATGATCCACG | *Fbp2*-distal-F |
| ZZZ48 | 5' GTAACCTCCTTCAGCTGGCC | *Fbp2*-distal-R |
| ZZZ49 | 5' CTACATCGTGGGTCTGCTCT | *CG31034*-common-F |
| ZZZ50 | 5' ATGCTGGCTCCGTAGTTGAT | *CG31034*-common-R |
| ZZZ51 | 5' AGTCCGTCGATGTCCAGATC | *CG31034*-distal-F |
| ZZZ52 | 5' CTTGCCTCCGTCAGTGTTGA | *CG31034*-distal-R |
| ZZZ53 | 5' GGAGAGGCGCCCTATATTGT | *CG11912*-common-F |
| ZZZ54 | 5' TCTGAACGTTCTCCTGGTCA | *CG11912*-common-R |
| ZZZ55 | 5' TCGGCCTAATTCTCCTCAAGG | *CG11912*-distal-F |
| ZZZ56 | 5' CCCAGCCGTACAGATATCCA | *CG11912*-distal-R |
| ZZZ57 | 5' CTGCCATGAGCTTTGGAGTA | *CG7597*-common-F |
| ZZZ58 | 5' AGTAGCGAAGCAAACAGCA | *CG7597*-common-R |
| ZZZ59 | 5' CTCCTACGGTGATAGCCTCG | *CG7597*-distal-F |
| ZZZ60 | 5' ACAGAATCCCTTATTGGTTACGT | *CG7597*-distal-R |
| ZZZ61 | 5' AGTGGCCAGTAACAAACGTG | *CG8878*-common-F |
| ZZZ62 | 5' CCGTGTTTGGGTTCAGTCTT | *CG8878*-common-R |
| ZZZ63 | 5' TCATACTTCGGACCTGTCGG | *CG8878*-distal-F |
| ZZZ64 | 5' CTATGGAGCTGAGAGTGCCA | *CG8878*-distal-R |
| ZZZ65 | 5' AGGACTCGAAGGCTGATGTG | *elF1A*-common-F |
| ZZZ66 | 5' CGAATGTGACTGTCTCGTTGA | *elF1A*-common-R |
| ZZZ67 | 5' TCCTGTTCGAAGTCATAGCCA | *elF1A*-distal-F |
| ZZZ68 | 5' AGGTTTGGTTTCGCAATTTAACTG | *elF1A*-distal-R |
| ZZZ69 | 5' CTCCCACCATCCGGCTGAACAAC | *CG9436*-common-F |
| ZZZ70 | 5' GTAGACGAAGGCGGTGTCCA | *CG9436*-common-R |
| ZZZ71 | 5' CACACGCGAGGAGGTTTTCGTGA | *CG9436*-distal-F |
| ZZZ72 | 5' TGTGCATCAGGTAGAGGTCT | *CG9436*-distal-R |
| ZZZ73 | 5' GAAGAACCGACACGACATGC | *CG18111*-common-F |
| ZZZ74 | 5' TCCACGTTGAAACCGCTCTG | *CG18111*-common-R |
| ZZZ75 | 5' AAGAACGAGCAGGGATCCAATG | *CG18111*-distal-F |
| ZZZ76 | 5' GCTTATCGTTACATCCGTCCAAC | *CG18111*-distal-R |
| ZZZ77 | 5' AGATTCTTCGCTGCAGCATG | *CG5861*-common-F |
| ZZZ78 | 5' CATCCAAGTCCAGCCGTTATG | *CG5861*-common-R |
| ZZZ79 | 5' ACCCTGATCTGGCTCTTCAC | *CG5861*-distal-F |
| ZZZ80 | 5' CCGTTAGCCAAGGTCCAATG | *CG5861*-distal-R |
| ZZZ81 | 5' TTGTGGATCTTACCGTCCGC | *Gapdh1*-F(qPCR for APA) |
| ZZZ82 | 5' GTGTAGCCCAGGATTCCCTT | *Gapdh1*-R(qPCR for APA) |
