# Supplementary material for: Deletions of singular U1 snRNA gene significantly interfere with transcription and 3’-end mRNA formation
Source: PLoS Genet. 2023 Nov 2;19(11):e1011021. doi: 10.1371/journal.pgen.1011021 (PMC10645366; doi:10.1371/journal.pgen.1011021)
Supplement: S6 Table — (DOCX) [file pgen.1011021.s016.docx]

**Wang & Liang S6 Table**

**S6 Table. *Drosophila melanogaster* strains used in this study**

| **Genotype** | | **Source** | | **Number** |
| --- | --- | --- | --- | --- |
| [*w^1118^*](http://flybase.org/reports/FBal0018186) | [Bloomington](https://bdsc.indiana.edu/) | | BDSC#5905 | |
|  |  | |  | |
| [*w^1118^*](http://flybase.org/reports/FBal0018186)*; U1:21D-gfp* | [this](https://bdsc.indiana.edu/) study | | N.A. | |
| [*w^1118^*](http://flybase.org/reports/FBal0018186)*; U1:82Eb-gfp* | [this](https://bdsc.indiana.edu/) study | | N.A. | |
| [*w^1118^*](http://flybase.org/reports/FBal0018186)*; U1:95Ca-gfp* | [this](https://bdsc.indiana.edu/) study | | N.A. | |
| [*w^1118^*](http://flybase.org/reports/FBal0018186)*; U1:95Cb-gfp* | [this](https://bdsc.indiana.edu/) study | | N.A. | |
| [*w^1118^*](http://flybase.org/reports/FBal0018186)*; U1:95Cc-gfp* | [this](https://bdsc.indiana.edu/) study | | N.A. | |
|  |  | |  | |
| [*w^1118^*](http://flybase.org/reports/FBal0018186)*; U1: 21D^Δ/Δ^* | [this](https://bdsc.indiana.edu/) study | | N.A. | |
| [*w^1118^*](http://flybase.org/reports/FBal0018186)*; U1: 82Eb^Δ/Δ^* | [this](https://bdsc.indiana.edu/) study | | N.A. | |
| [*w^1118^*](http://flybase.org/reports/FBal0018186)*; U1: 95Ca^Δ/Δ^* | [this](https://bdsc.indiana.edu/) study | | N.A. | |
| [*w^1118^*](http://flybase.org/reports/FBal0018186)*; U1: 95Cb^Δ/Δ^* | [this](https://bdsc.indiana.edu/) study | | N.A. | |
| [*w^1118^*](http://flybase.org/reports/FBal0018186)*; U1: 95Cc^Δ/Δ^* | [this](https://bdsc.indiana.edu/) study | | N.A. | |
